# Synergy of Artificial SEI and Electrolyte Additive for Improved Performance of Silicon Electrodes in Li-Ion Batteries

**DOI:** 10.1021/acsaem.4c01862

**Published:** 2024-10-14

**Authors:** Łukasz Kondracki, Janne-Petteri Niemelä, Dominika Baster, Mario El Kazzi, Ivo Utke, Sigita Trabesinger

**Affiliations:** † PSI Center for Energy and Environmental Sciences, Paul Scherrer Institute, Forschungsstrasse 111, Villigen CH-5232, Switzerland; ‡ Laboratory for Mechanics of Materials and Nanostructures, Empa, Feuerwerkerstrasse 39, Thun CH-3602, Switzerland

**Keywords:** li-ion batteries, anodes, Si, alucone coating, MLD

## Abstract

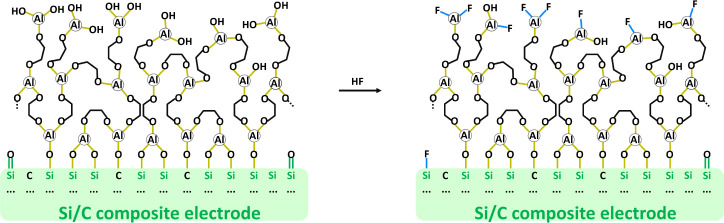

Maintaining the electrochemically and mechanically stable solid electrolyte interphase (SEI) is of highest importance for the performance of high-capacity anode materials such as silicon (Si). Applying flexible Li-ion permeable coatings to the electrode surface using molecular layer deposition (MLD) offers a strategy to improve the properties of the SEI and greatly contributes to an increase in the cycle life and capacity retention of Si electrodes. In this study, the long-term cycling of Si electrodes with an MLD alucone coating is investigated in the context of more stable SEI formation. When the joined strategy introducing both MLD coating and anFEC electrolyte additive was realized, high performance of Si anodes was achieved, capable of delivering more than 1500 mAh g^–1^ even after 400 cycles. The reason for the significantly improved longevity is the ability of the alucone layer to react with HF present in LiPF_6_-based electrolytes already under OCV-like conditions, fluorinating most of the available −OH groups in the alucone structure. This reaction not only partially scavenges hydrofluoric acid but also does not disturb the confining effect of alucone-like fluorinated artificial SEI. This study shows the significance of searching for synergetic solutions, such as a combination of electrode surface modification and electrolyte composition, for maximizing the capacity retention of Si as an active material or as a capacity-enhancing additive to graphite electrodes, and as well can be applied to other high-energy battery materials with large volume changes during cycling.

## Introduction

The increasing consumer demand for lighter, more durable, and longer-lasting Li-ion batteries is driving worldwide research toward improving the gravimetric and volumetric energy density of commercial battery cells. Battery performance is fundamentally limited by the choice of the electrode active materials for both the positive and negative electrodes. For the latter to contribute toward an increase in the energy density of battery cells, materials with specific capacities higher than those of graphite have to be used, preferably not sacrificing too much of the potential window. Therefore, Si has long been considered one of the most attractive solutions due to its high theoretical specific capacity (3579 mAh g^–1^), nearly 10 times higher than that of currently used graphite and its relatively low working potential.^1,2^ In practice, cell performance, especially when considering negative electrodes, depends on the solid–electrolyte interface (SEI) formation and its stability because they function outside the stability window of commonly used liquid electrolytes. An ideal interphase should be mechanically stable and electronically insulating while being ionically conductive.^3^ In the case of graphite, SEI formed upon the first Li-ion intercalation into the graphite via electrolyte reduction on graphite’s surface is close to an ideal one because most current battery electrolytes have been developed with graphite in mind. In contrast, the Si-based electrodes suffer from large volume expansion during alloying with Li,^4,5^ resulting in the cracking of the SEI layer upon cycling. Cracks cause continuous consumption of the electrolyte at the freshly exposed electrode surface, and as a consequence, a constant growth of the passivation layer is observed.^3,6^ Another implication of volume change is the loss of electrical contact between the active material particles and additives that improve conductivity and the current collector. These phenomena result in continuous overpotential buildup and rapid capacity fading of Si-containing electrodes.

The most popular strategy to improve the cycling stability of Si-anodes is the use of electrolyte additives with a higher reduction potential than the electrolyte solvents.^7,8^ Fluoroethylene carbonate (FEC) is the electrolyte additive of choice when it comes to enhancing the cycle life of Si and Si–graphite anodes.^9−12^ Lower charge transfer resistance is linked to the thinner surface film formation in the presence of FEC,^13^ and to the different chemical composition of SEI layers, facilitating Li^+^ transport.^14,15^ It has been reported that flexible polymers are the main beneficial components of the SEI layer formed in the presence of this additive.^13−15^ However, recently, we found that the products of fluoroethylene-carbonate decomposition first deposit as LiF spherical particles, and only then a continuous carbonate-rich film, covering the entire electrode, is formed.^16^ These LiF spheres are not observed on electrodes when using an FEC-free electrolyte consisting of only typical electrolyte components, such as ethylene carbonate (EC), diethyl carbonate (DEC), and LiPF_6_ salt.

The other approach is to try mimicking the SEI by prefabrication of an additional thin surface layer that shields the electrode components from the liquid electrolyte, even before the cell is assembled. This is often referred to as an artificial SEI (a-SEI). This treatment can be performed at two levels: on the powder level, where only the particles of the active material are coated (the most common approach), and on the electrode level, where the coating is applied to an already-prepared composite electrode.^17−19^ Among film coating techniques, molecular layer deposition (MLD) has emerged as one of the techniques that can produce uniform and conformal thin films, both inorganic–organic and purely organic, directly from the gas phase on complex surfaces with atomic precision.^17,20,21^ Given the degree of volume expansion of Si particles, surface coatings should be flexible to accommodate these, while remaining chemically stable. Here, the aluminum-based organic–inorganic hybrids (alucones) have received particular attention because of their high elasticity.^22−27^ So far, for the MLD-treated electrodes, performance-related research has focused mainly on the use of one type of electrolyte: either only carbonate-based electrolytes,^25,26,28^ or FEC-additive-containing electrolyte.^29,30^ The latter approach often attributes improvements in electrode performance solely to the coatings. Moreover, different Si mass loadings, electrode compositions (Si:C:binder ratio), and additive concentrations (FEC concentration varying from a few up to 20%_wt._) reported in the literature make the results hard to compare and difficult to understand their significance, as the mass loadings of electrodes are often omitted in the discussion. However, if the impact of the sacrificial additive is studied, this parameter becomes the battery cycle-life-determining factor.^31^ In addition, both the FEC-derived SEI and the alucone coating are expected to provide a flexible Si-confining layer, improving the lifetime and performance of the electrodes. Therefore, it is necessary to examine the properties of the two types of SEIs separately to distinguish their contributions and clarify their individual influence on cell performance and whether a synergistic effect can be achieved.

Therefore, in this study, the influence of alucone coatings and the FEC additive on the cycle life of Si anodes was clarified both in combination and individually. First, a range of different aliphatic alcohols were used as precursors for MLD-derived alucone coatings, which were then tested in half-cells with carbonate electrolyte without additives in order to assess the effect of alcohol chain length on cell performance and suitability for the a-SEI role. Then, the tests were conducted in the presence of FEC to discriminate the individual contributions of the coating and the additional benefits of the electrolyte additive. To better understand the working mechanism of this synergy and the final beneficial a-SEI composition, an X-ray photoelectron spectroscopy (XPS) surface study of the model electrodes, before and after being exposed to FEC containing electrolyte for both coated and uncoated variants, was performed.

## Experimental Section

### Materials

Si nanoparticles (30–50 nm, >98%) were purchased from Nanostructured and Amorphous Materials Inc. (Houston, USA). The conductive carbon SuperC45 was provided by Imerys Graphite and Carbon. Sodium salts of carboxymethylcellulose (CMC–Na) and lithium foil (750 μm, >99.9%) were purchased from Alfa Aesar. Glass fiber separators (EUJ 116, Hollingsworth and Vose, UK) were dried prior to use at 120 °C overnight under vacuum.

Trimethyl aluminum (TMA; Sigma-Aldrich 97%) was used as the Al precursor for the MLD coatings. The aliphatic alcohols used for introducing different organic ligands into the a-SEI were 1,2-ethanediol (i.e., ethylene glycol (EG); Sigma-Aldrich ≥99%), 1,6-hexanediol (HD; Sigma-Aldrich 99%), and 1,10-decane diol (DD; Sigma-Aldrich 98%).

The electrolytes used for cycling were LP30 (1 M LiPF_6_ in EC:DMC, v/v = 1:1, Gotion) and LP30–FEC (comprising 96 wt % LP30 and 4 wt % fluoroethylene carbonate, FEC, obtained from BASF). The LC30 (1 M LiClO_4_ in EC:DMC, v/v = 1:1) electrolyte for the immersion test was obtained from Gotion.

### Electrode Preparation

The electrodes contained Si, SuperC45, and CMC-Na in a mass ratio of 8:1.2:0.8. Deionized water was used as a slurry medium. A detailed description of the electrode preparation is provided elsewhere.^32^ Because the capacity fading of Si electrodes is mass-loading dependent,^31,33−36^ the loading was controlled within a close range of 1.4–1.7 mg_Si_ cm^–2^.

### Cell Assembly and Cycling Procedure

The dried electrodes were tested in half-cells using a coin-cell-type setup.^37^ Lithium was punched into discs of 13 mm diameter and used as a counter and reference electrode. The glass fiber separators were soaked by 500 μL of electrolytes. A constant and reproducible stack pressure in the cells was provided by the spring and closing of the cells using a torque wrench. The cells were conditioned during the first cycle with a slow C/25 (1C = 3579 mAh g^–1^) constant current–constant potential (CC–CP) charge and discharge cycle, with potential cutoffs of 5 mV and 1.5 V and a current cutoff of C/50). In subsequent cycles, the charging current was increased to C/10 maintaining the the same potential cutoffs but with a current cutoff of C/25).

### MLD Coating

The surfaces of the Si nanoparticle electrodes were coated by alucone thin films using the MLD. The coatings were deposited at 130 °C in a laboratory-built ALD/MLD reactor, and apart from the electrodes, planar Si substrates were coated for the thickness reference measurements. The depositions were carried out by cyclic dosing/purging of TMA and aliphatic alcohols. The EG precursor was heated to 80 °C, and the HD and DD precursors were heated to 105 °C for sufficient volatility; TMA was maintained at room temperature. Following our previous study on the process characteristics of these MLD coatings,^27,38^ the dose/purge times for TMA and the organic precursors were fixed to 0.15 s/120 s for EG/TMA, and to 6 s/240 s for HD/TMA and DD/TMA, respectively, with Ar as the purging gas. To ensure sufficient time for the precursors to diffuse into the open porosity of the nanoparticle electrodes, an exposure step was implemented in the deposition process.^39^ For this, a valve between the reactor chamber and the vacuum pump was closed before each precursor dose and then reopened 5 s after the end of the precursor dose.

### Reflectometry

For the XRR measurements, a Bruker D8 Discover diffractometer was used. The incident beam (Cu Kα) was conditioned by a Göbel mirror, a 0.1° divergence slit, and a 0.1° antiscatter slit. Measurements were done in θ–2θ geometry over a 2θ range of 0.1–5°, and the reflectivity patterns were analyzed using DIFFRAC LEPTOS (Bruker) software.

### Surface Characterization

The electrode morphology was investigated using scanning electron microscopy (SEM). A Carl Zeiss UltraTM 55 instrument. equipped with Everhart–Thornley (ETD) and Through-Lens (TLD) detectors, was used with a primary electron beam energy of 2 keV. XPS measurements were conducted with a VG ESCALAB 220iXL spectrometer (Thermo Fisher Scientific) using focused monochromatized Al Kα radiation (1486.6 eV) with a beam size of ∼500 μm^2^ (power, 150 W). The pressure in the analysis chamber was approximately 2 × 10^–9^ mbar. The spectrometer was calibrated on a clean silver surface by measuring the Ag 3d_5/2_ peak at a binding energy (BE) of 368.25 eV with a full-width at half-maximum (fwhm) of 0.78 eV. All spectra were recorded at a pass energy of 30 eV, in steps of 50 meV, and a dwell time of 50 ms. All the spectra were calibrated by setting the hydrocarbon/C–C C 1s peaks to a binding energy of 284.8 eV and processed by the Casa XPS software,^40^ using the Shirley-type background. Quantification was performed on the basis of Scofield’s photoionization cross-sections.^41^

All cells were prepared in duplicates to ensure reproducibility and account for mass discrepancies resulting from difficulties in the exact quantification of the nanosized Si loading. For the study, a relatively thick Cu current collector with a typical mass of 23.80(10) mg per electrode disc was used, while the obtained loading of Si was 1.4–1.7 mg_Si_ cm^–2^. Since the current collectors are significantly heavier than the other electrode components, this can result in significant errors in the active electrode mass estimation This problem is known for Si electrodes and has been previously reported.^36,42^ Nevertheless, the obtained standard deviation for each type of examined electrode is quite low, and the resulting small systematic error allows for drawing quantitative conclusions. All results are included in the Supporting Information for verification.

## Results and Discussion

### Coatings and Morphology

The coatings were applied on the already cast electrodes in order to protect all electroactive surfaces, accessible to electrolyte, and minimize electrolyte decomposition upon contact. This approach also potentially subjects an a-SEI to less stress, as the expansion of the electrode is lower than that of individual particles, and in addition, it does not disrupt the electronic network of the electrode.

It is known from the literature that 4 nm is a suitable thickness for alucone-based a-SEIs on Si nanoparticle electrodes.^25,26^ Therefore, the TMA/EG, TMA/HD, and TMA/DD processes, as specified by Niemelä et al.,^27^ were run for 9, 8, and 8 cycles, respectively, to target a thickness of 4 nm. The deposition cycle numbers were estimated from X-ray reflectometry thickness measurements of slightly thicker (∼10 nm) films deposited on Si-wafers. The XRR data and a typical XRR pattern are found in Table S1 and Figure S1. It is expected that the organic monomer units are bound together via O–Al–O linkages, so the material consists of repeating units of O–Al–O–(CH2)_*x*_–O–Al, where *x* (≥2) is the number of carbon atoms in the organic precursor. Ex-situ FTIR measurements also observed hydroxyl groups in the alucones.^27^

**Figure 1:**
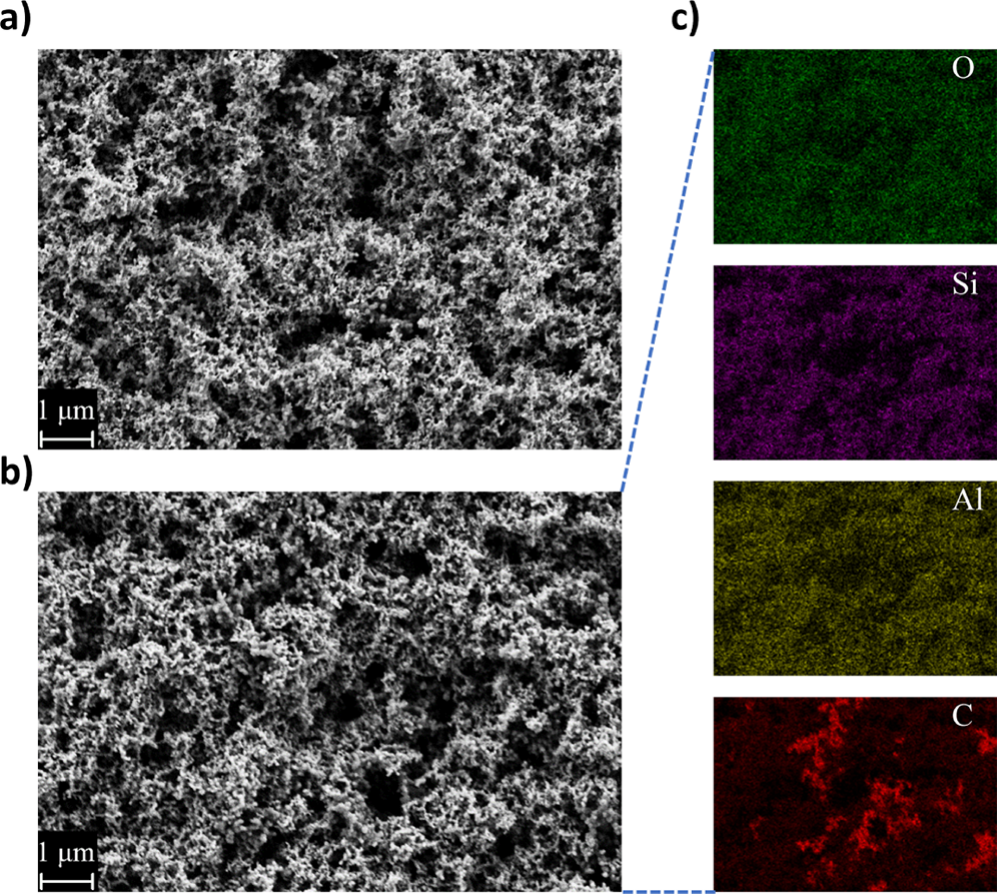
Scanning electron micrographs of Si electrodes: uncoated (a) and TMA/EG-coated (b) recorded at 2 kV. (c) EDX mapping of the elements detected on the TMA/EG-coated electrode.

In Figure 1, the images of the electrode surfaces, both uncoated (a) and TMA/EG-coated (b), are shown. There is no visible morphological difference between the uncoated and coated samples; the thin layer of alucone on the surface is not discernible. The EDX spectra (Figures S2 and S1) and maps show a homogeneous distribution of Al all over the surface of the electrode for all the coated samples (Figures 1c, S3, S4 and S5). In this energy range, clear signals from Si, oxygen, and carbon can be detected. The O and C signals can be attributed to the binder, carbon additive, and native SiO_*x*_ layer present on the Si nanoparticles. A small Na signal that is visible for the uncoated sample (Figure S13), originates from the CMC–Na binder. The TMA/EG-coated electrode exhibits an additional Al peak that can be assigned to the alucone coating. Additional proof of the coating presence is that the Na signal associated with the binder is not detectable in the presence of the coating.

### Performance of Uncoated and Coated Si Electrodes in LP30 Electrolyte with and without FEC

**Figure 2:**
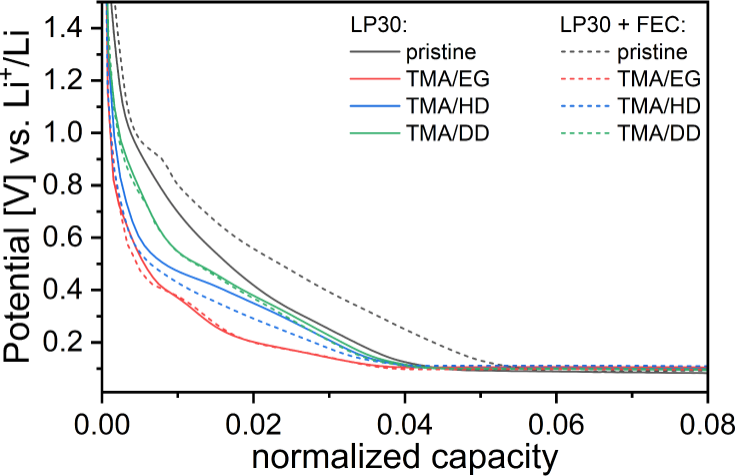
Voltage profiles of the first charge of Li|LP30|Si and Li|LP30+FEC|Si half-cells, with standard LP30 electrolyte (solid lines) and LP30 with FEC (dashed lines).

**Figure 3:**
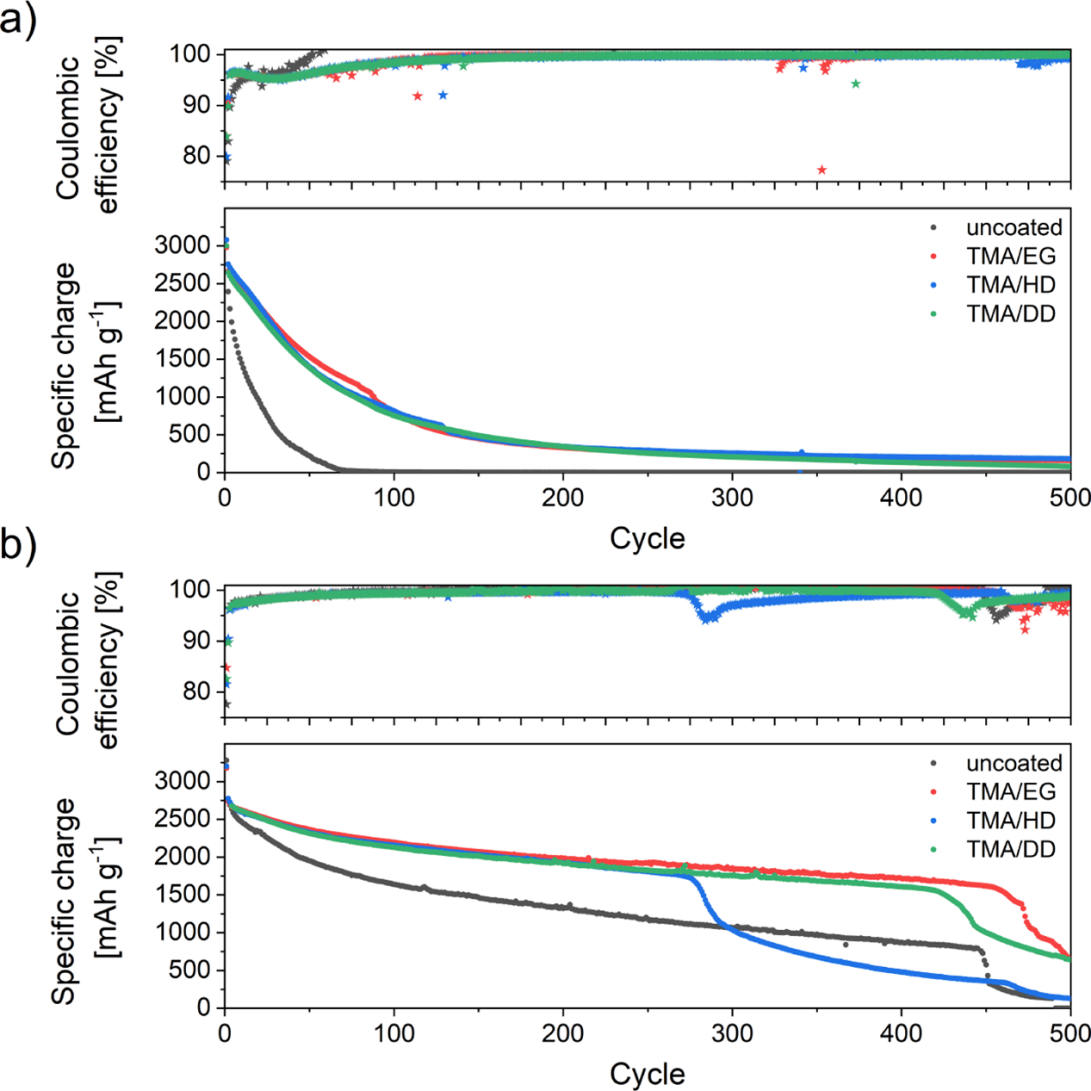
Galvanostatic cycling of coated and uncoated Si electrodes with LP30 (a) and LP30+FEC (b) electrolyte. First cycle is at C/25, and consecutive cycles at C/10. For clarity, only the best performing cells are shown. Comparison of all cells in the SI.

The comparison of the normalized potential profiles during charging is shown in Figure 2, where the lowest irreversible charge losses were recorded for TMA/EG-coated electrodes (160 mAh g^–1^), while in the case of TMA/HD- and TMA/DD-coated electrodes, they were very similar, of approximately 185 mAh g^–1^. The uncoated electrodes, cycled in the LP30 electrolyte, exhibit slightly higher irreversible charge loss of 200 mAh g^–1^. All the coated electrodes exhibit faster potential decay in the characteristic SEI formation region than the uncoated one, while the differences between each type of coating observed in terms of capacity retention were not very significant (taking into consideration the difficulties in handling and weighing the electrodes with Si nanoparticles). However, a clear dependence between the SEI formation potential profile during the first charge and the type of aliphatic alcohol used for MLD is clear: the denser the alucone film, the faster the decay of the potential (see Table S1), indicating fewer side reactions at a given potential value before reaching the Si lithiation potential. Upon charge, the voltage decays the fastest for the TMA/EG-coated samples, and then TMA/HD, TMA/DD, following the trend in coating density values, and finally the uncoated electrodes. This indicates that density can be a measure of a better quality of the a-SEI, preventing electrolyte degradation, as well as correlating with capacity retention during the first 80 cycles (Figures 3 and S5.)

For the cells cycled with the LP30+FEC electrolyte, the comparison of the normalized charge curves of the initial cycle is shown in Figure 2 with dashed lines. A plateau at a potential of around 0.95 V vs Li/Li^+^ (black dashed line) is in the range of the FEC decomposition potential,^16,43,44^ and is prominent only for the uncoated Si electrodes, resulting in an irreversible capacity loss during the first cycle of 250 mAh g^–1^ (50 mAh g^–1^ more than in LP30 without FEC, indicating sacrificial reduction of the additive). It is worth noting, especially for the MLD-coated electrodes, that the irreversible capacity loss for the coated electrodes is the same for both tested electrolytes. All three types of coated electrodes exhibit nearly overlapping potential profiles in both standard LP30 and FEC-enriched LP30 electrolyte, with a slight deviation in the case of TMA/HD, which has a somewhat higher overpotential in LP30+FEC than in LP30. Furthermore, the same correlation between the density of the a-SEI and the rate of potential decay is observed also for the FEC-containing electrolyte. The similarity of the potential profiles and the lack of characteristic plateau around 0.95 V vs Li/Li^+^ indicate that FEC does not play a major role in SEI formation during the initial cycle for the MLD-coated electrodes and that coatings are preventing FEC decomposition, but not EC and DMC reduction at lower potentials. Again, with exception for TMA/HD, which seems to suppress slightly more the reduction of EC and DMC with the addition of FEC (d*Q*/d*E* curves of data from Figure 2 presented in Figure S6).

The cycling performance of Si half-cells with the LP30 electrolyte for the first 200 cycles is presented in Figure 3a, along with the corresponding Coulombic efficiencies. In the case of all three coatings, it is clear that improvement in capacity retention has been gained as compared to the uncoated Si electrodes. The discharge capacity of the first cycle (C/25) for all of the tested electrodes was comparable, slightly above 3000 mAh g^–1^. During the following cycles (C/10), the capacity of uncoated electrodes deteriorated rapidly, falling below 1000 mAh g^–1^ already in the 19th cycle, while for the coated electrodes the capacity retention improved significantly–it took nearly 80 cycles for all the MLD-coated electrodes to reach the same value of capacity fade. This behavior has been confirmed by the duplicate cells (Figure S7).

Importantly, the MLD coatings also improved the Coulombic efficiency, especially the first cycle efficiency of the cells. The cells with uncoated Si electrodes exhibited an initial CE of 80%. For all MLD-coated samples, it was improved by 5% for the initial cycle, and then stabilized at around 95% already after two cycles, while the cells with uncoated electrodes reached this value only after 12 cycles. This indicates that the SEI formed during the first three cycles for MLD-coated electrodes is clearly more stable than that of the uncoated ones.

Figure 3b presents results of cycling the best-performing Si half-cells with uncoated and coated electrodes using LP30+FEC as electrolyte. In this case, the performance of each cell is presented separately in Figure S8, because of the discrepancies in long-term performance, even among electrodes that underwent the same treatment. This behavior was not observed for cells cycled in LP30 without FEC, where the error bars are relatively small (compare with Figure S7).

**Figure 4:**
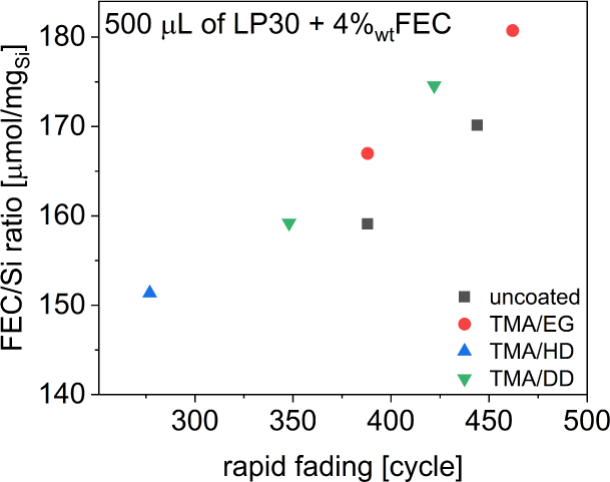
FEC/Si ratio vs rapid fading plot for all the tested cells with LP30+FEC electrolyte.

As expected, the addition of FEC greatly improved the performance of the cells for the uncoated electrodes. The FEC helps rebuild the SEI during consecutive cycles as a sacrificial additive, allowing cells with uncoated electrodes to reach more than 350 cycles with capacities exceeding 1000 mAh g^–1^. This improvement is also reflected in the Coulombic efficiency, increasing from slightly below 80% in the first cycle to more than 95% in the third and subsequent cycles. The cells with uncoated electrodes start to fail between 388 and 444 cycles, as indicated by a rapid drop in Coulombic efficiency (Figure S8a) and sudden capacity fade. All cells with coated electrodes and LP30+FEC electrolyte significantly outperformed the baseline, delivering a specific charge above 2000 mAh g^–1^ even after 270 cycles, and in some cases, even after 450 cycles. The difference in performance, regardless of the type of coating, is again not visible, similarly to the cells without the FEC additive. The Coulombic efficiencies of the initial cycle are notably higher (82–85%) than those of the cells with uncoated electrodes; however, from the second cycle, there are almost no differences. Also, for the coated electrodes, a similar failure behavior can be observed, manifested by a drop in Coulombic efficiency and rapid capacity fade. Again, large differences in cell life can be registered, even for the electrodes with the same type of coating. To address this phenomenon, an examination of the number of cycles of each cell and comparison of the numbers against the mass loading of each electrodes was carried out. A linear correlation was found between rapid fading and Si loading, regardless of the type of coating (Figure 4). Additionally, even uncoated Si electrodes in the presence of LP30+FEC electrolyte follow a similar trend of rapid capacity fading after prolonged cycling. This indicates that FEC depletion seems to be the main reason for the failure mechanism,^31,45,46^ This claim is supported by the behavior of the electrodes in cells with LP30 without FEC (Figures 3a and S7), where differences between the electrodes with the same type of coating and different loadings displayed only minimal performance differences.

**Figure 5:**
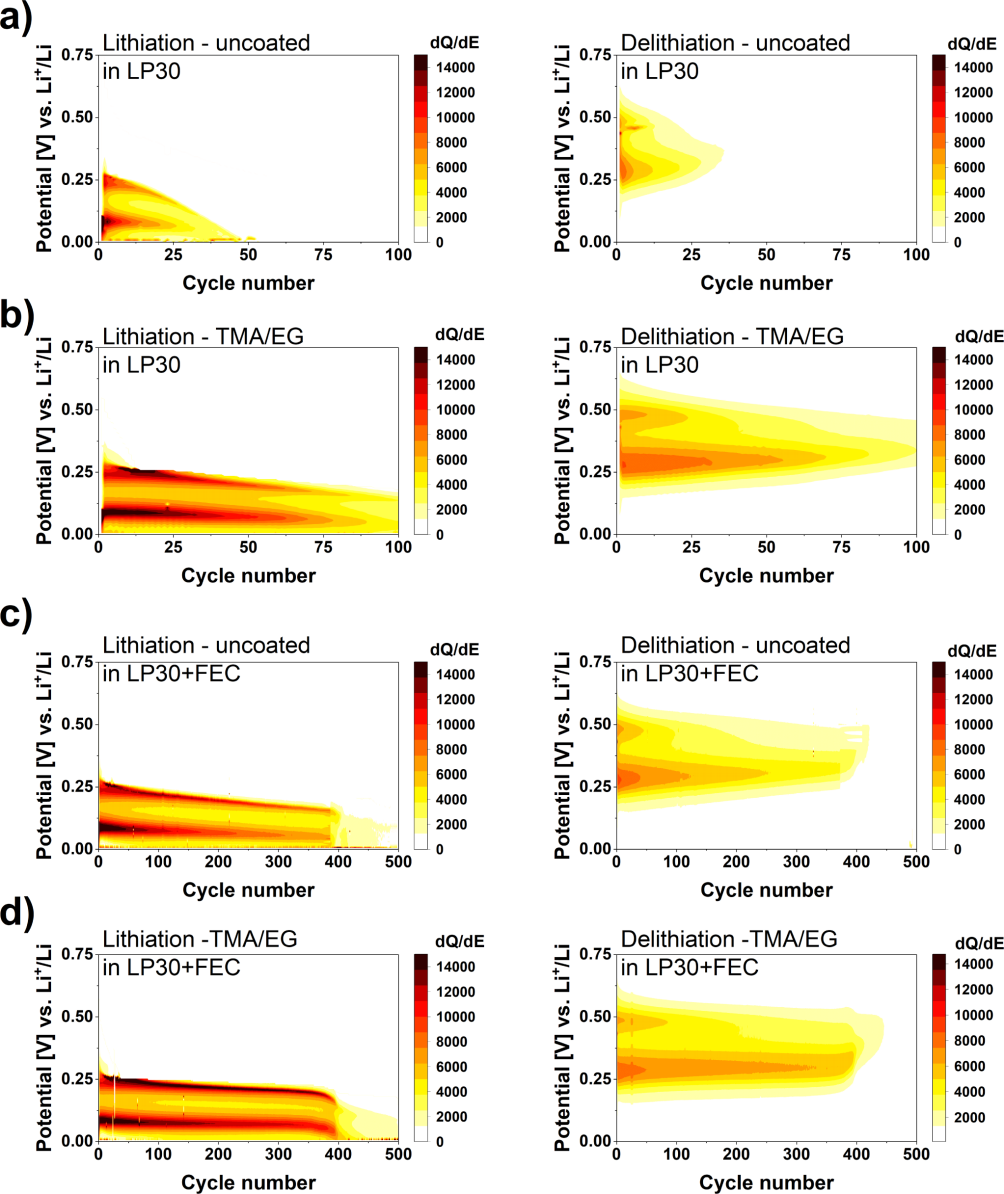
Heat maps of d*Q*/d*E* vs potential and cycle number for lithiation and delithiation of uncoated Si electrode (a, c) and TMA/EG-coated electrode (b, d).

To understand the effects of the coatings on the electrochemical performance, heat maps of the differential capacity and d*Q*/d*E* were analyzed. The heat maps for uncoated electrodes and TMA/EG-coated are shown in Figure 5, for both LP30 and LP30+FEC electrolytes. This plot provides a continuous picture of the electrode degradation process (a description of translating d*Q*/d*E* plots of selected cycles to d*Q*/d*E* heat maps is shown in Figure S9). The heat maps of other cells are shown in Figures S10 – S17. Color intensities reflect the peak intensity: the more intense the colored area at the respective potential, the higher the values for d*Q*/d*E* are. In all heat maps, we can see that the lithiation of all the tested materials is a two-step process. During the first lithiation step (at a potential of 0.25 V vs Li/Li^+^), an amorphous phase of lithiated Si alloy (a-Li_*x*_Si) is formed.^32^ The second plateau below 0.1 V vs Li/Li^+^ is attributed to the formation of Li-richer alloys, including the crystalline Li_15_Si_4_ phase.^47^ In the initial delithiation cycles, the conversion of these phases into a-Li_*x*_Si is represented by the plateau at 0.25 V vs Li/Li^+^, while the plateau corresponding to the reconversion to amorphous Si is located at 0.45 V vs Li/Li^+^. In the case of all the studied materials, a lowering of the lithiation potential of the first reaction (aSi → aLi_*x*_Si) with the prolonged cycling is clearly visible, moving from the initial values of 0.25 to 0.125 V vs Li/Li^+^. This behavior can have two possible explanations,^32^ first one being overpotential buildup from SEI growth, and the second being incomplete delithiation in the cycle before. It can be seen that the plateau corresponding to the delithiation of amorphous a-Li_*x*_Si alloys (around 0.45 V vs Li/Li^+^) decays even faster indicating incomplete reconversion to the amorphous Si. It can be seen from Figure 5 that the peaks of the cells with the uncoated electrode decay very fast. The lowering of the lithiation potential of the first reaction with consecutive cycles of the cell is very rapid, moving from the initial values of 0.25 to 0.125 V vs Li/Li^+^ within roughly 25 cycles. The plateau corresponding to the delithiation of the amorphous Li_*x*_Si alloy disappears almost completely after 12 cycles. This asymmetry between the existence of plateaus existence indicates that Li–Si alloys are not fully reconverted to the amorphous Si. It can be seen that the specific charge delivered by the cell comes mainly from the plateau assigned to the delithiation of Li-richer phases, especially after the other delithiation plateau disappears.

In contrast, MLD-coated Si electrodes (Figures 5b, and S10 – S13) show a similar type of plateau behavior as that for the uncoated samples, however, with a significantly longer participation in the characteristic alloying reactions of Si and Li. The first lithiation plateau (Si to Li_*x*_Si) is visible for over 80 cycles and displays much slower overpotential buildup than for the cell with an uncoated Si-electrode. The delithiation plateau of this phase is visible for 40 cycles, compared to 12 cycles in uncoated electrodes. However, as in the case of uncoated electrodes, the coated electrodes, the second plateau (delithiation of Li-richer phases) delivers most of the capacity as well.

All the tested coatings exhibit nearly identical thicknesses (Table S1), varying mainly in the densities of the obtained alucone. However, the lack of influence of the latter parameter on performance and the comparison with the electrochemical performance of the electrodes (testing the cells with different MLD coatings) indicate that the aliphatic carbon precursor used for the alucone production was of secondary importance.

For the comparison of voltage evolution between uncoated and coated electrodes in cells with LP30+FEC electrolyte, two cells with nearly the same rapid fading onset were chosen, namely, those that lasted nearly 390 cycles. The heat maps for the uncoated electrode and TMA/EG-coated are shown in Figure 5c,d, (Figures S14 – S17 present a comparison of heat maps of all the tested cells with LP30+FEC electrolyte). During the formation of an amorphous phase of lithiated Si alloy (first lithiation step), the difference between uncoated and TMA/EG-coated electrodes are rather quantitative; much more Si is lithiated in this phase in cells with the coated electrodes, which is reflected in the darker shade on the heat maps. The lowering of this lithiation potential with continuous cycling is not as rapid as for uncoated electrode in LP30 (Figure 5a) until the onset of rapid capacity fading (full consumption of the available FEC) starts. The formation of Li-richer alloys below 0.1 V vs Li/Li^+^ is also analogous to the first step, with significantly more Si being lithiated in the coated electrodes. The differences in performance in LP30+FEC are more visible upon delithiation. For the uncoated electrode, the delithiation of the amorphous alloy (0.45 V vs Li/Li^+^) disappears after nearly 50 cycles (12 cycles for LP30, Figure 5a), whereas for the TMA/EG-coated electrode, it is present up to 100 cycles (40 cycles for LP30 without additive). For both compared here electrodes the specific charge delivered by the cell comes mainly from the plateau assigned to the delithiation of Li-richer phases, which again is presented with a darker shade for TMA/EG-coated electrode. Similar to the LP30 electrolyte, there is an asymmetry between plateau existence, indicating that Li–Si alloys are not fully reconverted to the amorphous Si.

### Coating Reactivity with Electrolyte

XPS was used to investigate the role that the FEC-enriched electrolyte plays in SEI formation during the initial cycle of the MLD-coated electrodes. The scope of the study has been narrowed down to the comparison of the uncoated and TMA/EG-coated electrodes since the differences between the initial charge curves were more pronounced in these two cases. Moreover, as shown above, different densities of the alucone layers had no significant effect on the long-term performance of the cells. The uncoated and TMA/EG-coated electrodes were immersed into LP30+FEC electrolyte solution for 24 h. Then, after the samples were washed with DMC, XPS spectra were recorded for both soaked and unsoaked samples. The quantification of elements found on the surface of the electrodes is presented in Table 1, calculated based on the survey scans of the electrodes (Figure S18).

**Table 1:** Quantification of the Elements Detected on the Surface of the Examined Electrodes

	Atomic %			
Element	uncoated	uncoated + LP30+FEC	coated	coated + LP30+FEC
Si 2p	44.7	36.3	6.3	5.3
C 1s	20.0	16.2	39.4	18.6
O 1s	27.3	20.7	27.6	14.8
F 1s	5.0	16.4	13.7	38.2
Na 1s	1.2	-	-	0.2
Li 1s	-	9.6	-	13.8
Al 2p	-	-	10.6	9.0

**Figure 6:**
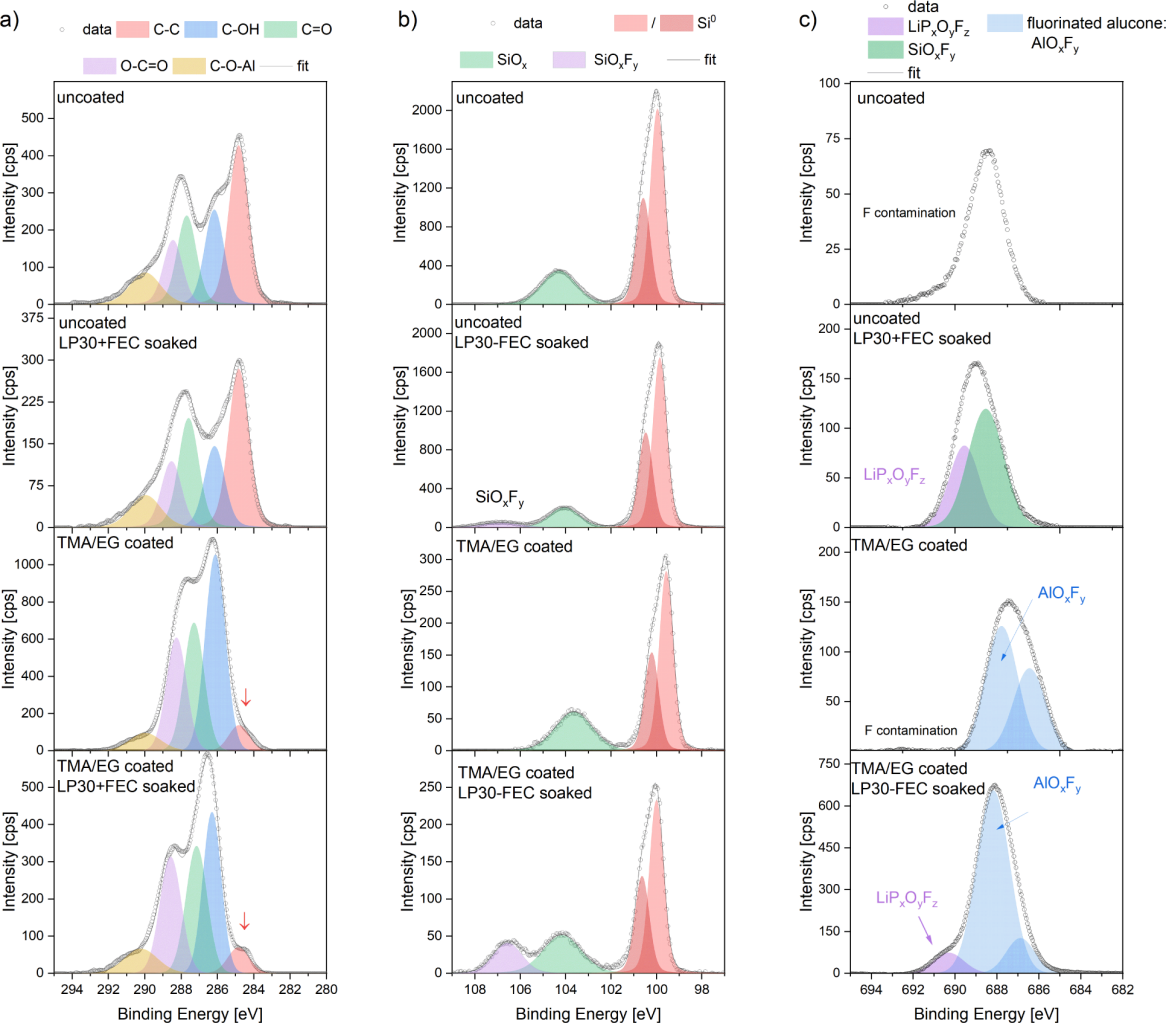
C 1s, (a) Si 2p, (b) and F 1s (c) XPS core levels acquired on Si electrodes, both unsoaked and immersed in LP30+FEC electrolyte.

The C 1s core level peaks (Figure 6a) for uncoated and soaked electrodes can be deconvoluted into a few components, with a major one at 284.8 eV, associated with the C–C bond. The other distinct peaks at 286.2, 287.7, and 288.5 eV (C–O, C=O, and O–C=O, respectively) originate from the mono- and bioxygenated environments of carbon in CMC-Na binder. The deconvoluted peak at 289.9 eV can be attributed to the adsorbed carbon species CO_3_^2–^, which has already observed on the surfaces of air-exposed samples.^48,49^ The fitting parameters are provided in Table S2.

For the alucone-coated samples, the carbon spectra show the most intensive peak at 286.1 eV (Figure 6a), which can be attributed to C–O–(Al/H) species, originating from the alucone coating.^27,50^ In addition, the deconvoluted peak for the C–C type bonds decreases significantly in its intensity compared to uncoated samples (red arrow in Figure 6a), indicating that the signal assigned to Super C45 carbon additive is barely detectable by XPS (penetration depth of ∼10 nm), which is in good agreement with MLD thickness (Table S1).

The relative intensity of the peak attributed to the C–OH type bonds decreases (blue) after immersion of the electrode into the electrolyte, hinting at reactions between alucone and the electrolyte.

Figure 6b shows the XPS spectra of Si for the same samples. The Si 2p_3/2_ spectra of uncoated electrodes exhibit a main peak at 99.9 eV. A slight shift of all Si-related peaks toward lower binding energies for the coated electrode (99.5 eV) is visible also for SiO_*x*_ and for SiO_*x*_F_*y*_ type bonds. This phenomenon has been attributed to fluctuations in the surface charging effect of thin layers with different conductivity, coated on Si-substrates.^51,52^ Moreover, the Si 2p_3/2_ intensities point to the fact, that the outermost Si are barely detectable by the XPS, which is in a good agreement with the targeted MLD thickness calculated from XRR measurements and the C–C signal from Super C-45 (Figure 6a). The peaks attributed to SiO_*x*_ originate from the native Si oxide layer present in air-exposed Si. The formation of SiO_*x*_F_*y*_ on both electrolyte-immersed samples (both uncoated and coated) is a result of the reaction of the native Si oxide with hydrofluoric acid, HF,^49,53,54^ (product of LiPF_6_ hydrolysis, caused by water traces present in electrolyte):
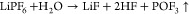
1
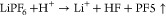
2
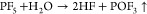
3

**Figure 7:**
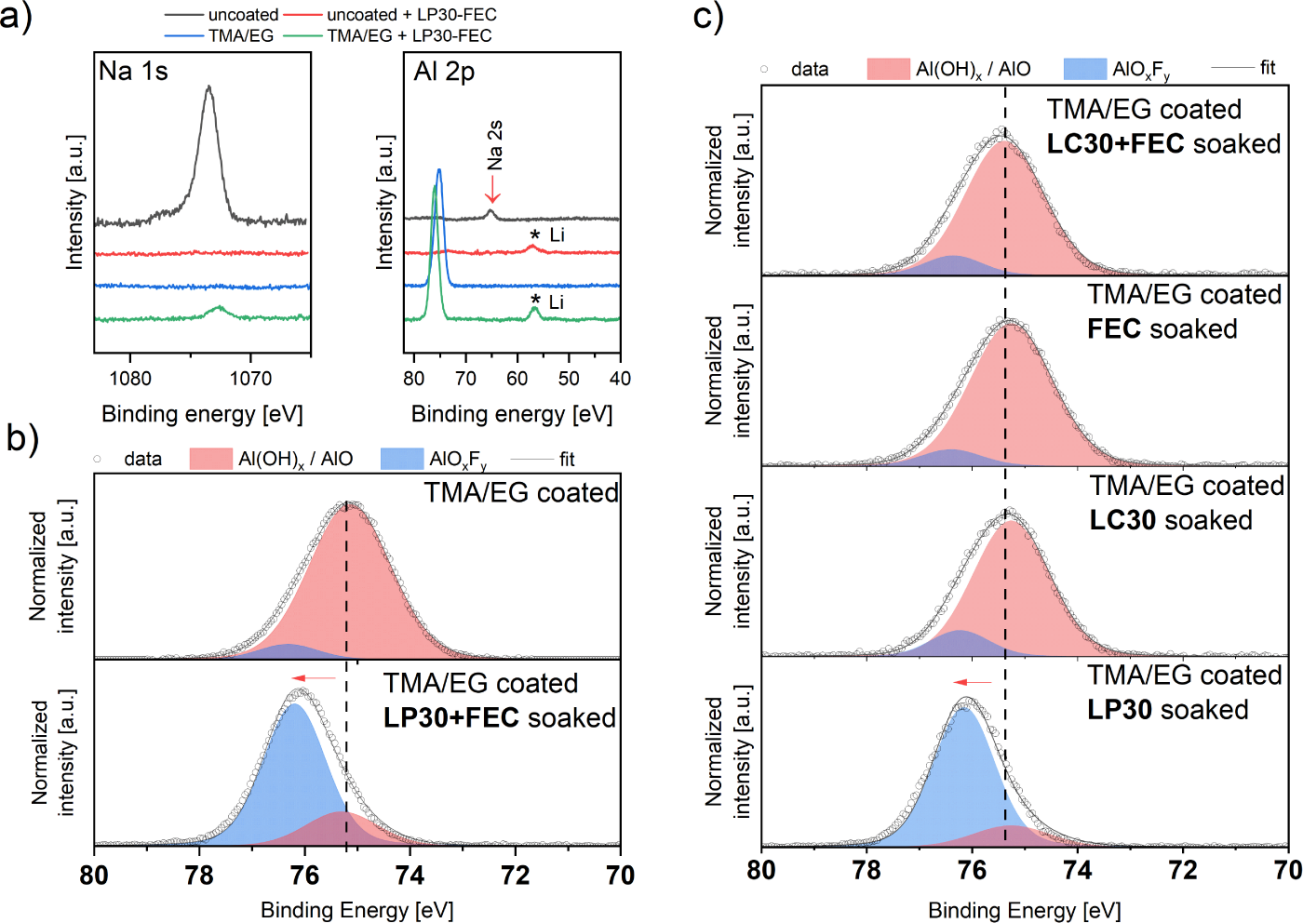
Na 1s, Al 2p, and Li 1s spectra (a). Deconvolution of Al 2p spectra (b) for TMA/EG-coated Si electrodes immersed in LP30+FEC (b) and immersed in LP30, FEC, LC30, and LC30+FEC electrolytes (c).

The spectra presented in Figure 7a show detectable Na for uncoated and coated + soaked samples, originating from the CMC–Na binder, used for the electrode preparation. The lack of Na peaks for the uncoated + soaked sample can be explained by binder particles being washed away by the electrolyte from the surface of the electrode or the exchange between electrolyte Li^+^ and binder Na^+^ ions.^55,56^ The coated sample also does not display this feature, which can be explained by the alucone layer successfully covering the examined area of the electrode to the degree that renders sodium untraceable. However, the presence of the Na signal for the coated + soaked sample shows that the Na-containing binder particles were washed from the layers underneath the coating and trapped within the alucone-free spaces. The alucone coating could also become thinner due to the dissolution in the electrolyte, but this is not the case, as the intensities of the Al peak do not change between the coated and coated + soaked samples. The peaks present at 57 eV, marked with asterisks in Figure 7a for the soaked samples, originate from residues of LiPF_6_ salt deposited on the surface of electrodes, soaked in the LP30+FEC electrolyte.

Aluminum hydroxides are difficult to differentiate by XPS, as the peak position for Al(OH)_3_ and AlO(OH) species vary in literature reports between 74.8 and 75.2 (with a standard deviation of 1 eV) and overlap with each other.^57^ The Al 2p spectra of MLD-coated samples shown in Figure 7b, are deconvoluted with two main components, broader one representing the aluminum hydroxide-like species (main expected component of the alucone coating^27^ with a binding energy of ∼75.2 eV and second one, 76.2 eV, that can be assigned to aluminum oxyfluorides (AlO_*x*_F_y_).^58,59^ The demonstrated change of these peaks’ area suggests fluorination of the alucone protecting layer already during the OCV-like conditions (Table S3).

This is consistent with the shape of the XPS F 1s spectra (Figure 6c and Table S4). The uncoated sample exhibits a peak of relatively low intensity, which can be attributed to contamination on the surface of the electrode. The source of this fluorine contamination in both the uncoated and TMA/EG-coated samples is most probably the glovebox atmosphere.^60^ The uncoated and LP30+FEC soaked sample exhibits significantly more intense and broader peaks, which can be deconvoluted into two main components. The first component at 689.6 eV (purple), which is in good agreement with the residues of LiPF_6_ salt deposited on the surface of the electrodes soaked in the electrolyte and the second one (green) at 688.6 eV can be assigned to SiO_*x*_F_*y*_ species (confirmed by Si spectra in Figure 6b).

The coated samples, however, display a shift in the peak position toward lower binding energies. In the case of these samples, the spectra can be deconvoluted into two broad main peaks at 688.0(4) and 686.7(3) eV. Both peaks can be assigned to AlO_*x*_F_*y*_ species^58,59,61,62^ (reports from the literature with a standard deviation of 1 eV). The peak at the lower binding energy can also be identified as partial fluorination of (CH_2_)_*x*_ links in the alucone coating.^63^ This possibility could not be excluded based on carbon spectra. Nevertheless, the increase in the intensity of the fluorine peak centered at a higher energy (688 eV) for the TMA/EG-coated + LP30+FEC soaked electrode suggests that this is the main AlO_*x*_F_*y*_ component. This observation, which is in good agreement with Al spectra presented in Figure 7b, confirms the hypothesis of alucone fluorination. The coated + soaked sample additionally exhibits one smaller peak from LiPO_*x*_F_*y*_ (690.3 eV). In this case, SiO_*x*_F_*y*_ component overlaps with peaks correlated to the fluorinated alucone layer, and due to small intensity, it can be only speculated by its appearance in Si spectra (Figure 6b).

These results allow us to formulate another question, namely, about the nature of alucone fluorination. There are two potential fluorine sources in the FEC-enriched LP30 electrolyte: the first one is LiPF_6_ salt (or more precisely, HF from the mentioned hydrolysis), and FEC. Fluoroethylene carbonate itself can lose an HF molecule to form a VC molecule, as shown in (reaction 4):^64−66^
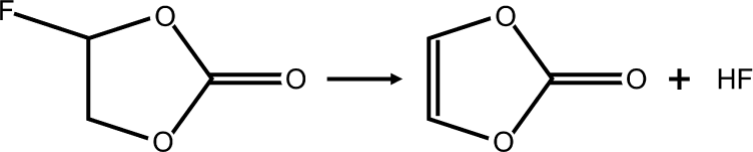
4

The detailed mechanism of FEC decomposition is still an open question. Recently, especially in theoretical studies,^67−69^ it has been claimed that fluorine is more likely to leave the FEC in the form of an F^–^ ion rather than HF molecule. Another observed reaction pathway in the presence of Si is the one in which reduced FEC releases the F^–^ ion, which bonds to hydrogen originally adsorbed on a Si anode surface.^13^ Moreover, an HF elimination pathway has also been suggested from FEC^–^ in the presence of EC solvent, but the calculations excluded the formation of VC as one of the products.^70^

To verify the origin of the fluorination of alucone, further immersion studies have been conducted. The TMA/EG-treated electrode was immersed in four different types of solutions: LP30, FEC, LC30, and LC30 + 4 wt % FEC (LC30+FEC) for a duration of 24 h. In the LP30 solution, the only possible fluorine source is HF from LiPF_6_ salt. LC30 is a 1 molar solution of LiClO_4_ in an EC/DMC mixture and does not contain any fluorine. A pure FEC solution would assign the fluorination of the alucone coating solely to FEC-derived fluorine, similarly to the FEC-enriched LC30 electrolyte. The quantification of XPS measurements is shown in Table S5 and the survey spectra in Figure S19. Comparison of C 1s, Si 2p, and Al 2p spectra of all the analyzed samples are presented in Figures S20 – S22.

Interestingly, only the electrode immersed in LP30 showed an increase in the amount of F species on the surface, approximately 34%, which is very close to the value obtained for FEC-enriched LP30 electrolyte (38%, Table 1). Immersion in pure FEC, LC30, and LC30+FEC solutions revealed nearly the same amount of fluorine on the surface as TMA/EG-coated electrode that had not been soaked (14 atom %, or below). The detailed analysis of the XPS Al 2p spectra for TMA/EG-coated Si electrodes immersed in LP30, FEC, LC30, and LC30+FEC solutions is presented in Figure 7c. Only the sample immersed in the LP30 electrolyte exhibited peak shifts toward higher binding energies, indicating fluorination of −OH groups. Upon deconvolution, a very similar AlO_*x*_F_*y*_:Al(OH) ratio is obtained compared to the sample immersed in LP30+FEC electrolyte (Figure 7b). The three remaining samples display spectra nearly identical to those of the sample that has not been soaked in any of the electrolytes (Figure 6c). This result clearly indicates that FEC does not participate in the initial fluorination of the alucone layer. Moreover, HF elimination from FEC is also excluded in OCV-like conditions. It seems that only HF from LiPF_6_ hydrolysis is responsible for the fluorination of Al(OH) species of alucones.

Even though there seems to be no consumption of FEC in the first cycle (Figure 2), the coated electrodes consume more FEC during consecutive cycles (Figure 4), which would indicate either cracking or dissolution of the coating. However, as the immersion tests show the presence of all the constituents of the coating, it is most likely the cracking of the a-SEI is the reason.

**Figure 8:**
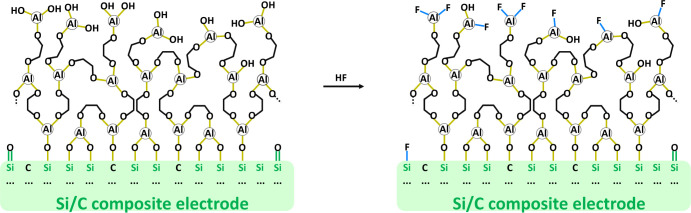
Scheme of TMA/EG obtained an alucone film on the surface of Si-electrode and proposed fluorination reaction upon LP30+FEC immersion.

Knowing that the alucone obtained during the MLD process, also consists of approximately 7% AlO_*x*_F_*y*_ species, an updated scheme of this coating is presented in Figure 8, describing the fluorination of the alucone/Si interphase. Immersion in a LiPF_6_-based electrolyte allowed us to fluorinate almost 80% of the Al(OH)_*x*_ species (Table S3). For the coated electrodes already at the OCV conditions, HF is consumed not only by the available Si/C electrode surface but also by the alucone layer itself.

This fluorinated alucone serves as a stable SEI during the initial charge. The reaction taking place between HF and the alucone layer leads to the modification of the artificially created solid electrolyte interphase on the surface of the Si electrode. The lack of a characteristic plateau, related to the decomposition of FEC (Figure 2) during the initial charge of cells with coated electrodes, can be attributed to the enhanced chemical stability of this new fluorinated alucone layer. This and the fact that the charge curves obtained during the first cycle for the LP30+FEC electrolyte are nearly identical to the ones obtained for the LP30 electrolyte without the additive suggest that FEC is being electrochemically activated only during consecutive cycles and in this system becomes more of a “healing” additive than a “SEI-forming” additive. The performance of the cells with coated electrodes in the LP30+FEC electrolyte point toward a synergistic effect of two types of SEI: artificial, fluorinated-alucone obtained via MLD and *in situ*-FEC-derived during consecutive cycles of the cells.

## Conclusions

A comparative study of long-term performance of alucone-coated Si electrodes in both pure LP30 and FEC-containing LP30 electrolyte was carried out. Alucone coating itself has been shown to positively affect the performance of Si electrodes, even in a standard LP30 electrolyte, which provides more than twice the lifetime. The dependence between the density of the obtained alucone and the shape of the first lithiation curve has been found; however, this does not correlate with the long-term cell performance.

As expected, the incorporation of FEC additive to the electrolyte composition considerably improved the performance of all the cells with both uncoated and coated electrodes. The differences in the performance of cells tested with the FEC-enriched electrolyte are closely related to Si loading, even for the MLD-treated electrodes. There is a linear dependence between the loading of the electrodes and the number of cycles, after which a rapid decrease in capacity occurs, indicating the consumption of all available FEC. Although the additive does not seem to contribute to the formation of SEI during the initial cycle in all of the MLD-treated electrodes, it plays a crucial role in performance improvement of the consecutive cycles. This means that the use of FEC shifts from SEI-forming to the “SEI-healing”, which means that the same amount of the additive will stay longer in the cell, as it is not consumed during the first cycle. Despite the slightly longer cycle life of uncoated electrodes in the presence of an electrolyte containing FEC, additional alucone coatings offer greater capacities and better capacity retention.

It was found that the alucone coating layer not only serves as confining barrier for Si-particles, but also scavenges HF present in LiPF_6_-containing electrolytes. This means that alucone coatings can reduce HF concentration in the cells; consequently, this reduction might affect cathode surface stability in full cells, and reduce metal leaching. The effects of the alucone-based a-SEI in full cells need to be still quantified in order to understand true significance of this finding.

A synergistic effect of alucone coating and FEC addition to the electrolyte shows a new way for the design strategies of Si-based anodes. This approach can be used for other electrode materials and in other areas where surface reactivity is an issue, contributing to the increasing energy density of future battery generations.

## Supporting Information Available

X-ray reflection studies, SEM images of the electrodes, extended electrochemical data, extended XPS quantification results (PDF)
