# Supplementary material for: Synergy of Artificial SEI and Electrolyte Additive for Improved Performance of Silicon Electrodes in Li-Ion Batteries
Source: ACS Appl Energy Mater. 2024 Oct 14;7(20):9336–48. doi: 10.1021/acsaem.4c01862 (PMC11523034; doi:10.1021/acsaem.4c01862)
Supplement: Supplementary file 1 — ae4c01862_si_001.pdf [file ae4c01862_si_001.pdf]

Supporting Information:

Synergy of Artificial-SEI and Electrolyte Additive for Improved Performance of Silicon Electrodes in Li-ion Batteries

Łukasz Kondracki [a,*], Janne-Petteri Niemelä[b], Dominika Baster[a], Mario El Kazzi[a], Ivo Utke[b], Sigita Trabesinger [a,*]

[a] *PSI Center for Energy and Environmental Sciences, Paul Scherrer Institute, Forschungsstrasse 111. CH-5232 Villigen PSI, Switzerland*

[b] *Laboratory for Mechanics of Materials and Nanostructures, Empa, Feuerwerkerstrasse 39, Thun CH-3602, Switzerland.*

*e-mail: lukasz.kondracki@psi.ch; sigita.trabesinger@psi.ch

1. MLD conditions

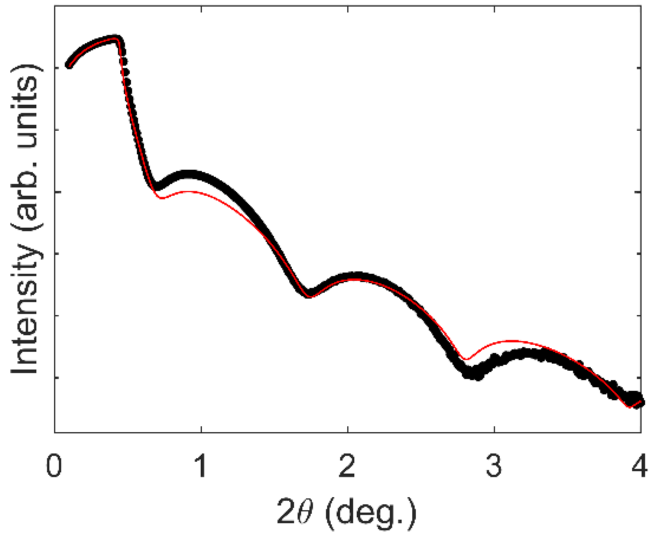

Figure S1. An example X-ray reflection pattern for the alucone film deposited through 17 TMA/EG MLD cycles; the data and the fit are shown with the black solid circles and the red line, respectively.

Table S1. X-ray reflection results for the alucone films deposited in the TMA/EG, TMA/HD and TMA/DD processes on flat Si substrates.

Process	Number of MLD cycles	Thickness (nm)	Roughness (nm)	Density (g/cm^3)
TMA/EG	17	8	0.6	1.71
TMA/HD	17	10	0.5	1.40
TMA/DD	17	9	0.6	1.34

2. Morphology of the electrodes

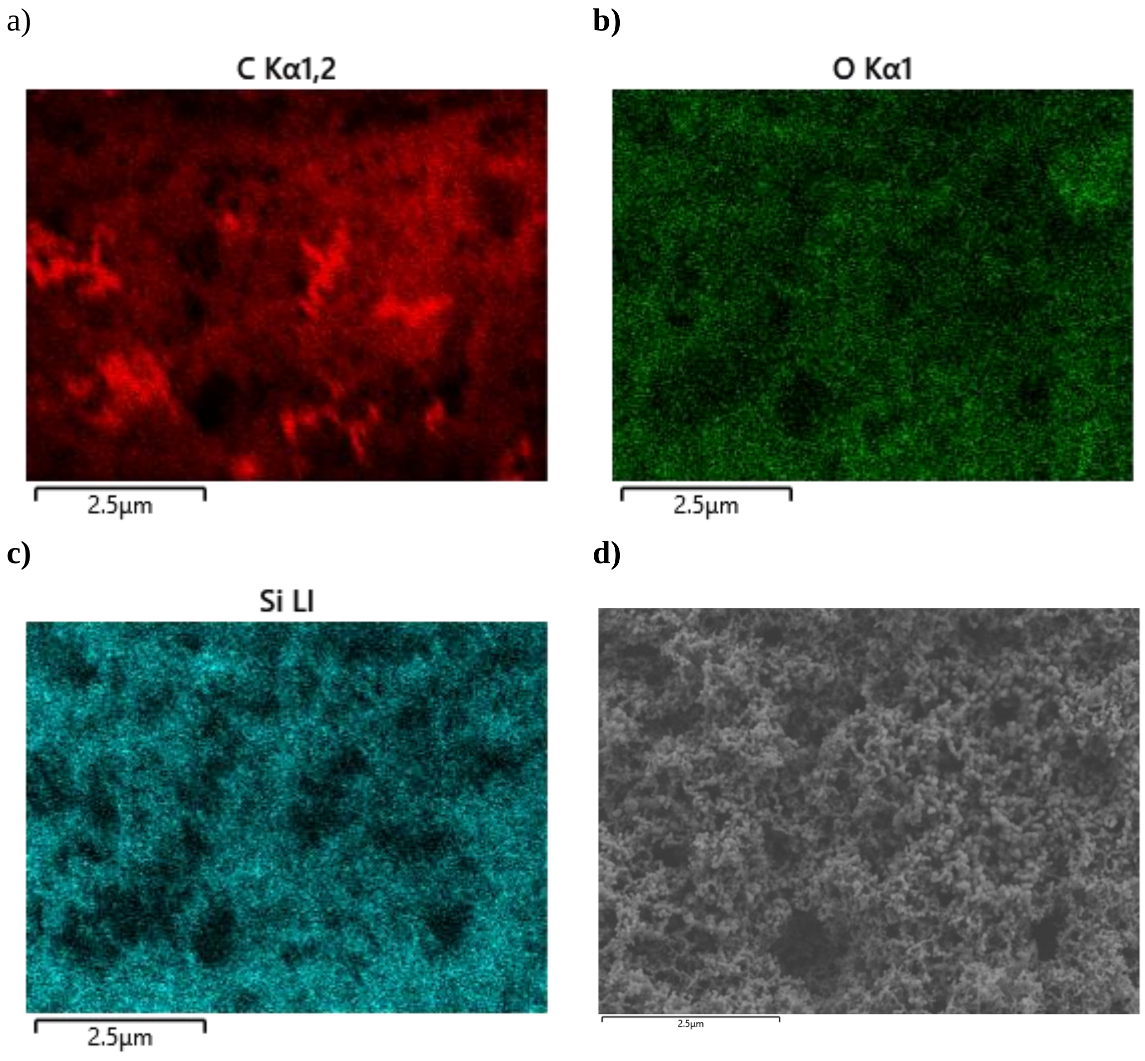

Figure S2. EDX mapping of the elements detected (a-c) on a non-coated electrode. SEM image of the mapped area for comparison (d).

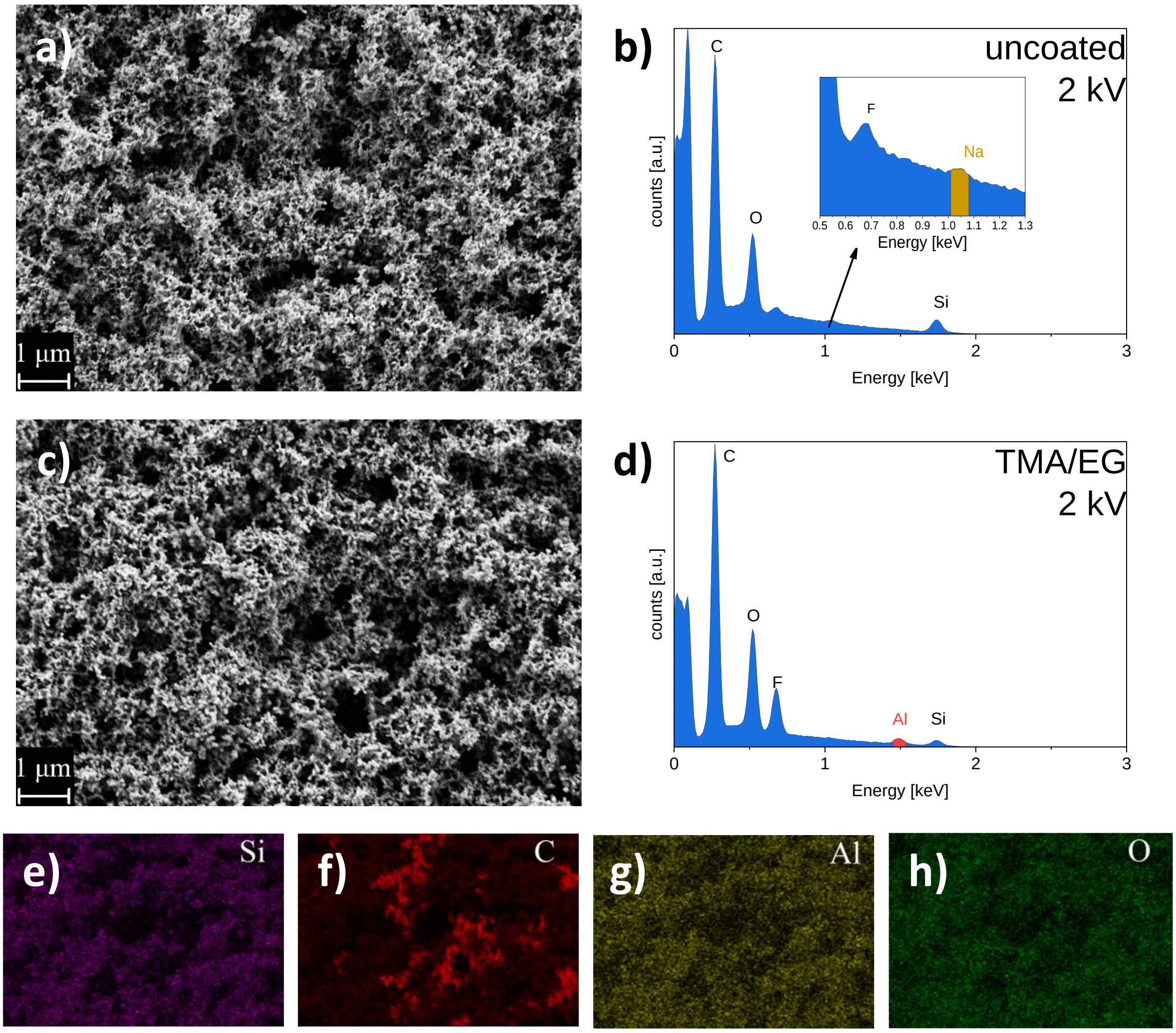

Figure S3. Scanning electron micrographs of Si electrodes: uncoated (a) and TMA/EG treated (c). EDX spectra of uncoated (b) and TMA/EG treated (d) electrode registered at 2kV. EDX mapping of the elements detected (e-h) on TMA/EG treated electrode (reference micrograph shown in c)).

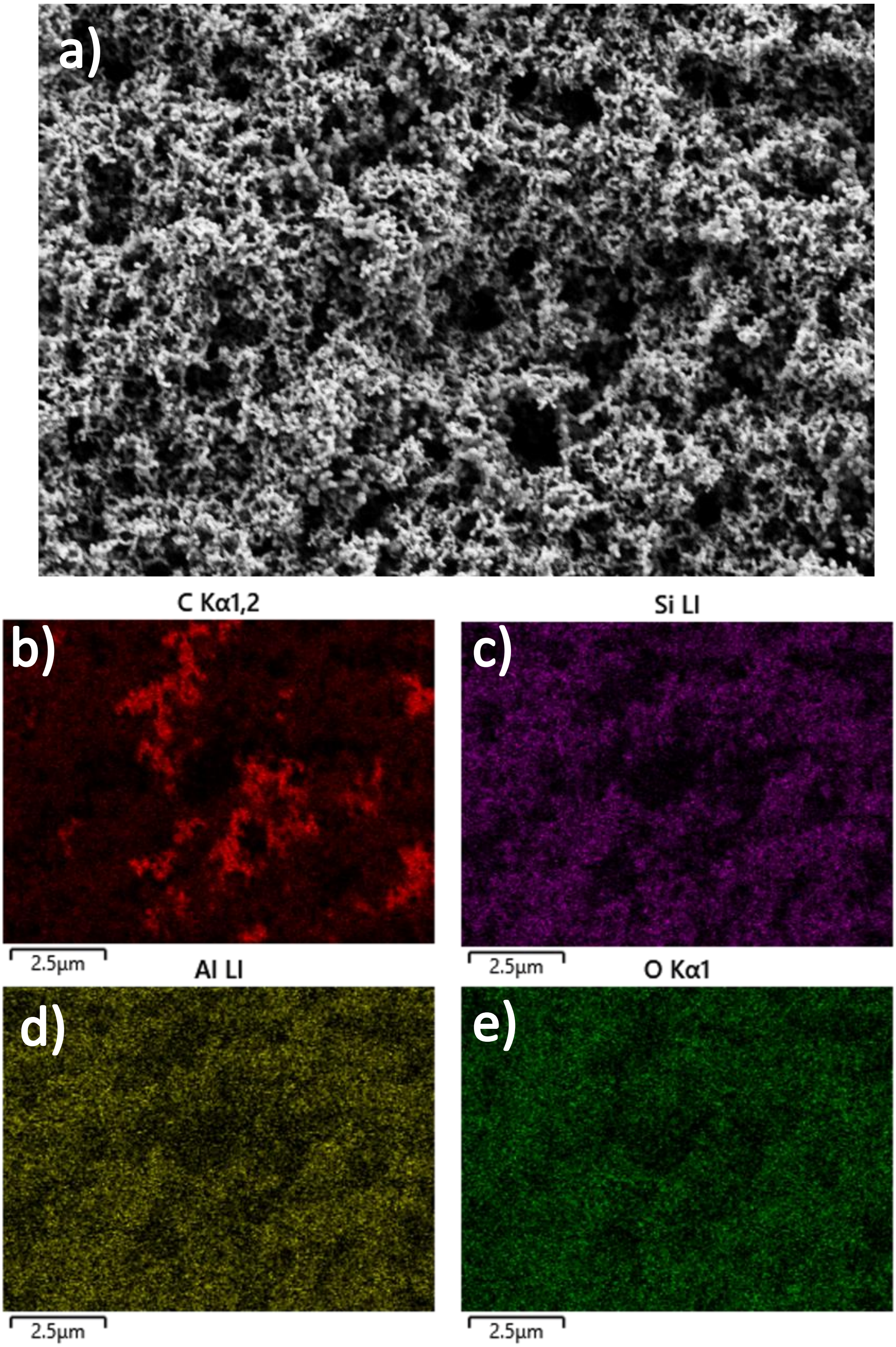

Figure S4. EDX mapping of the elements detected (b-e) on a TMA/HD treated electrode. SEM image of the mapped area for comparison (a).

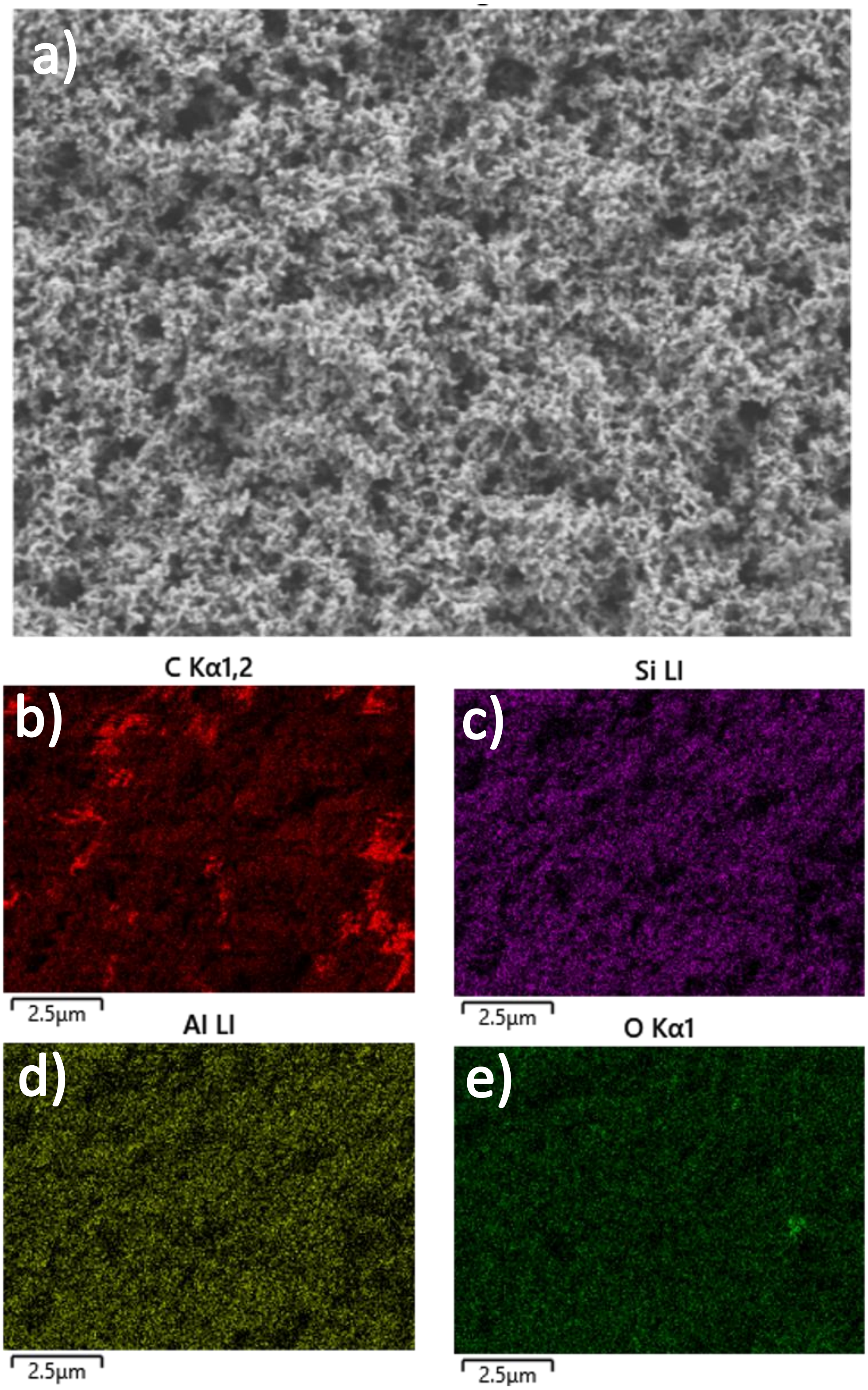

Figure S5. EDX mapping of the elements detected (b-e) on a TMA/DD treated electrode. SEM image of the mapped area for comparison (a).

3. Performance of Si electrodes in LP30 electrolyte and in LP30 + 4% FEC

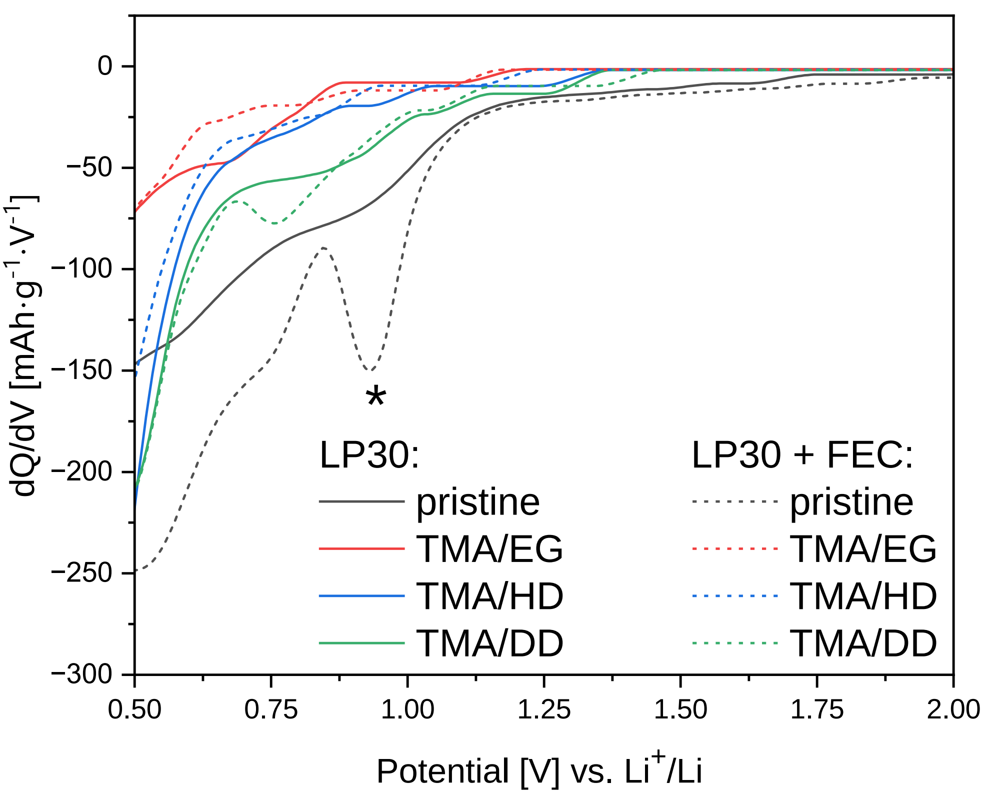

Figure S6. dQ/dV curves of the first charge of Li|LP30|Si and Li|LP30+FEC|Si half-cells with standard LP30 electrolyte (solid lines) and LP30 with FEC (dashed lines).

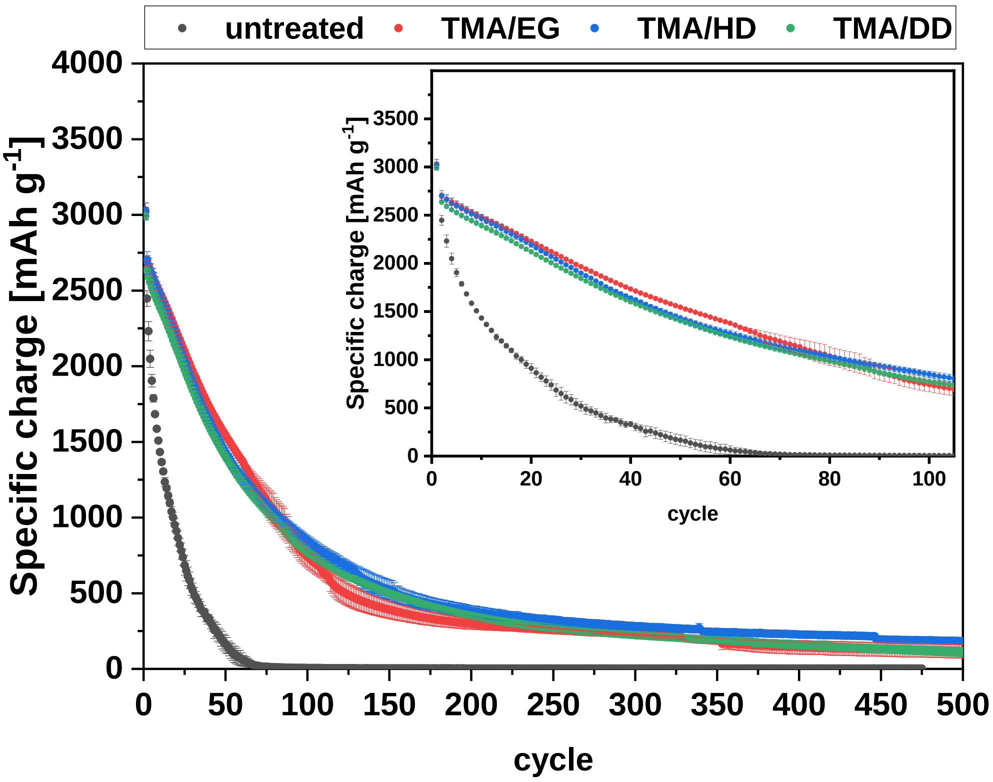

Figure S7. Electrochemical performance of Si electrodes examined in Li|LP30|Si half-cells: Long-term tests averaged over 2 cells for each electrode type. Enlargement of first 100 cycles for clarity (inset). No significant discrepancies in capacity retention between electrodes of the same type (coated nor pristine) were observed for the half-cells with LP30 electrolyte.

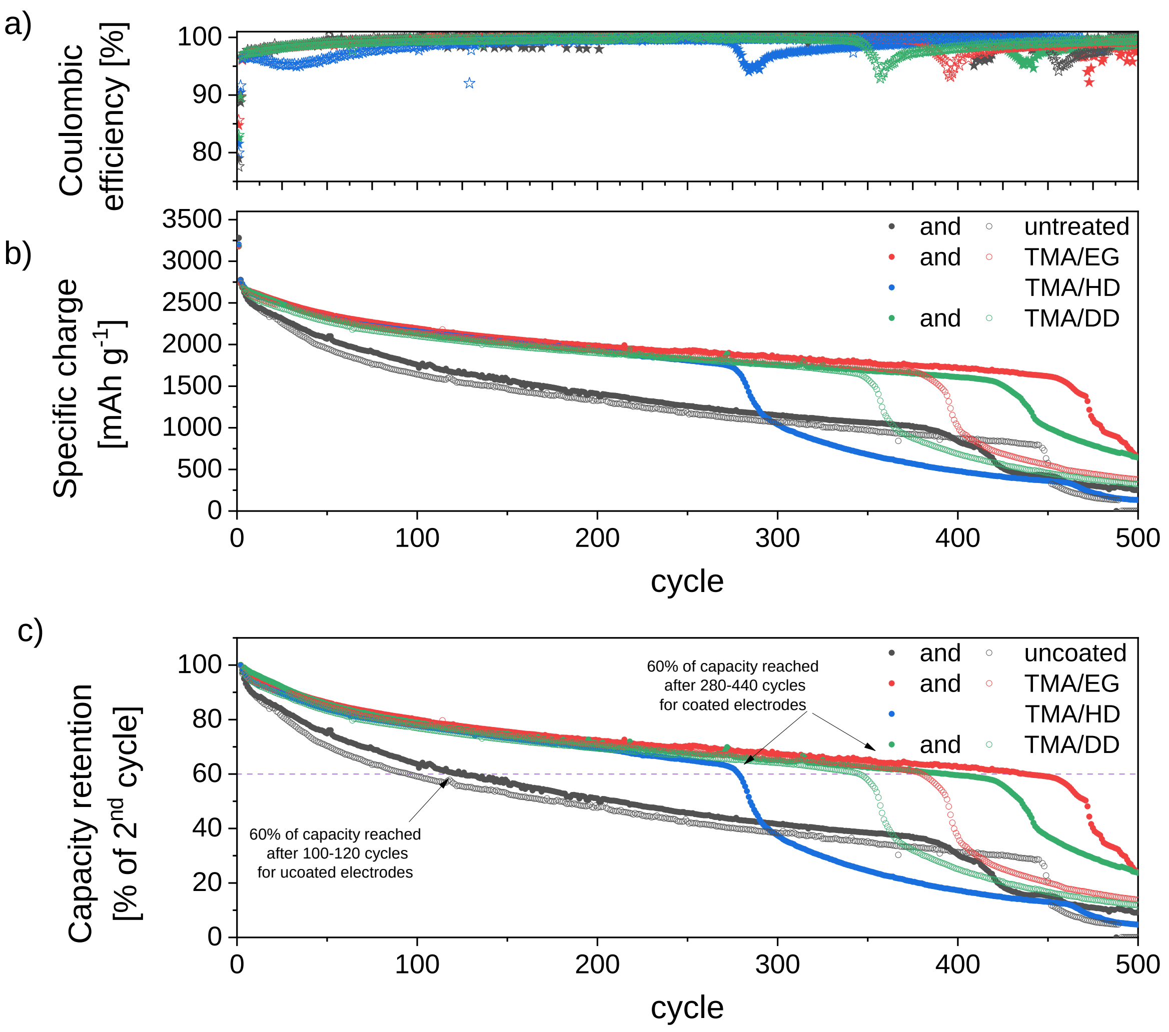

Figure S8. Galvanostatic cycling of coated and pristine Si electrodes with LP30 electrolyte with FEC additive. (a) Coulombic efficiency (b) First cycle C/20, consecutive cycles C/10. c) capacity retention compared to 2nd cycle .

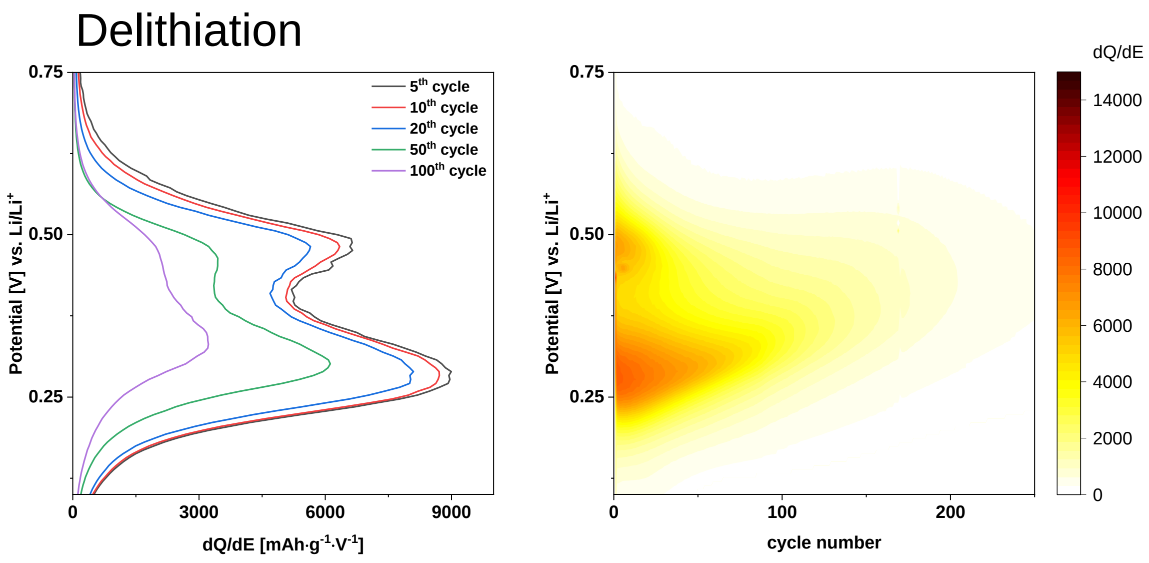

Figure S9. Example: From dQ/dE plots of selected cycles to dQ/dE heatmaps.

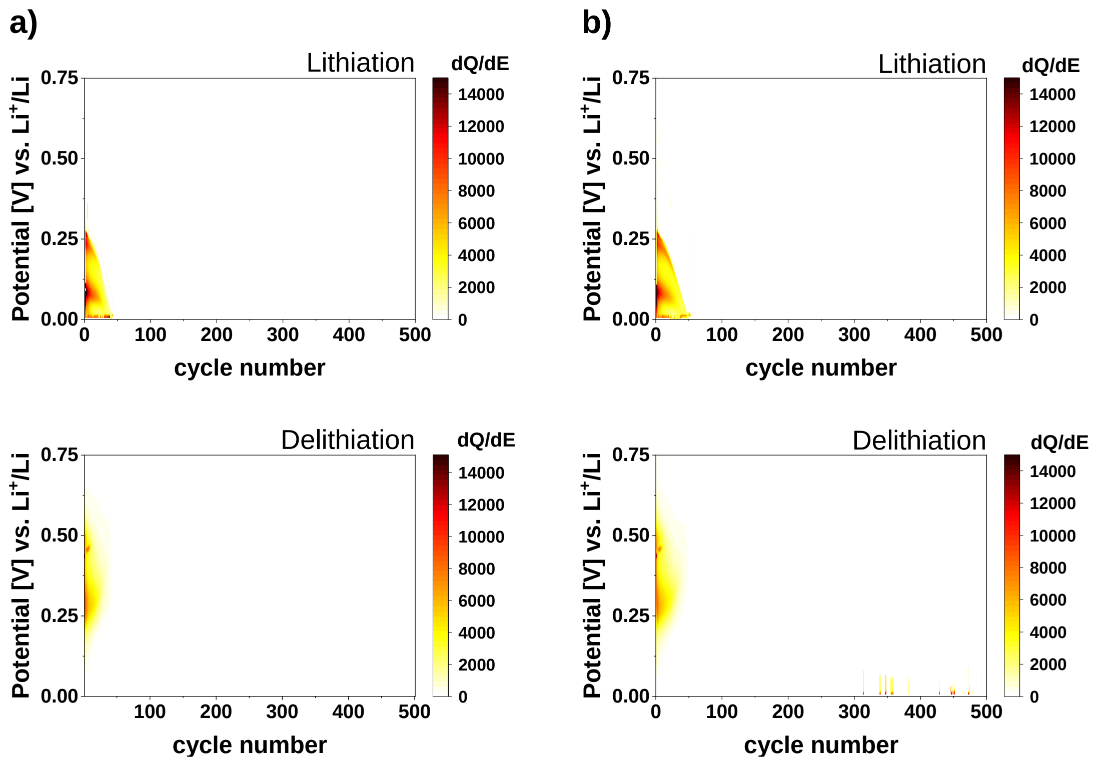

Figure S10. Heatmaps of dQ/dE vs. potential and cycle number for lithiation and delithiation of untreated Si electrodes with a loading of: 1.50 mg (a) 1.61 (b).

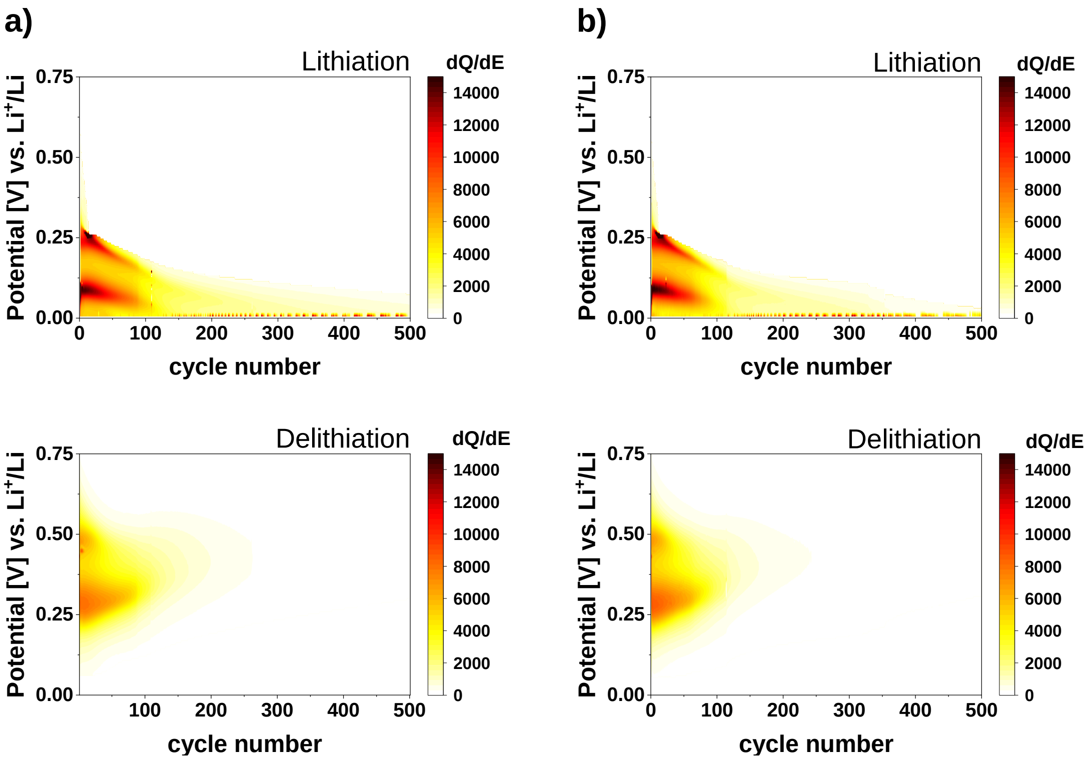

Figure S11. Heatmaps of dQ/dE vs. potential and cycle number for lithiation and delithiation of TMA/ethane-1,2-diol treated electrodes with a loading of: 1.39 mg (a) 1.40 (b).

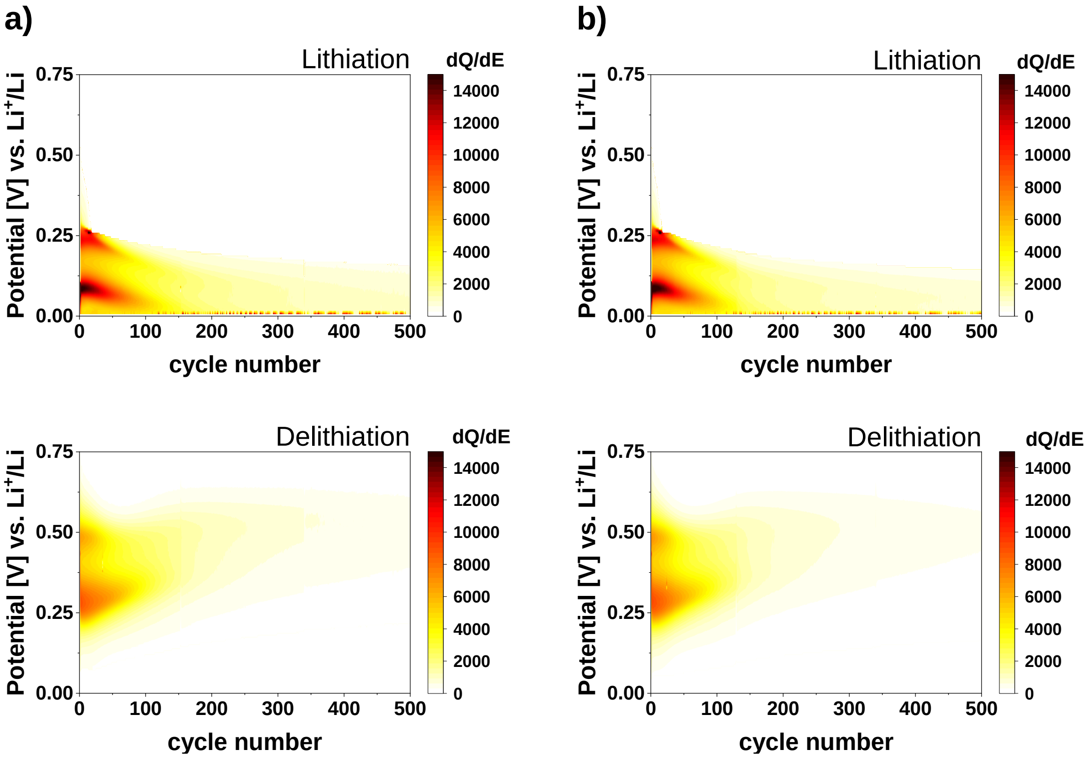

Figure S12. Heatmaps of dQ/dE vs. potential and cycle number for lithiation and delithiation of TMA/hexane-1,6-diol treated electrodes with a loading of: 1.69 mg (a) 1.62 mg (b).

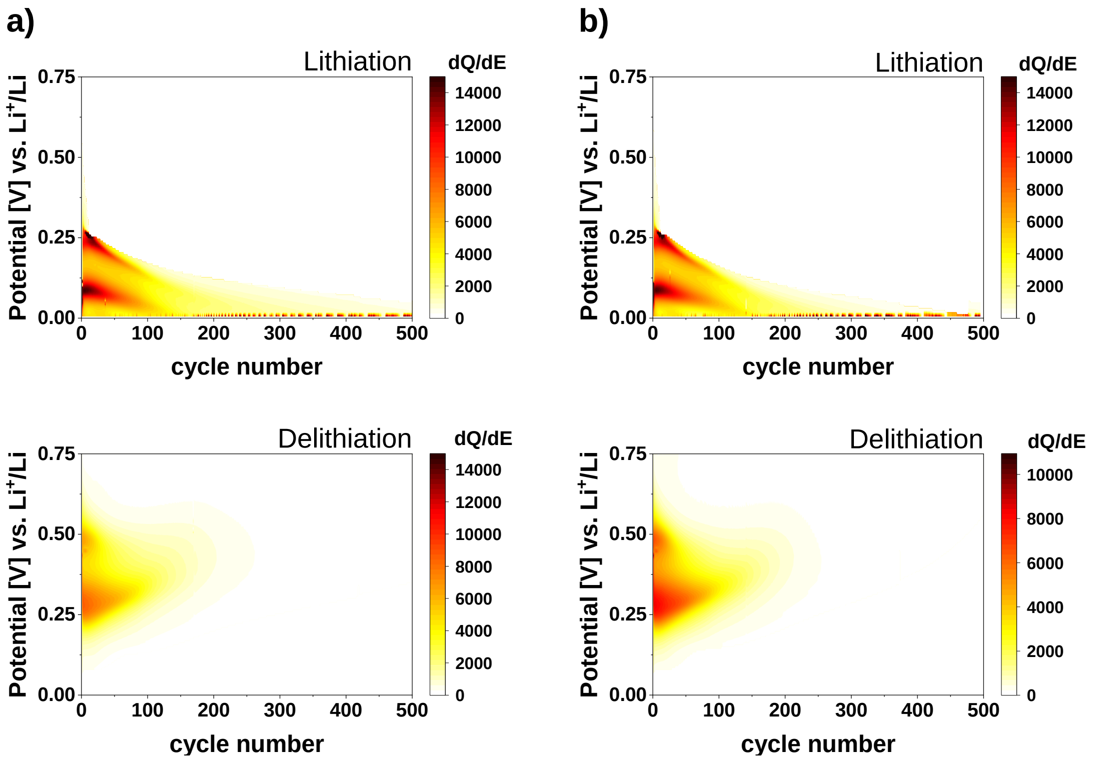

Figure S13. Heatmaps of dQ/dE vs. potential and cycle number for lithiation and delithiation of TMA/decane-1,10-diol treated electrodes with a loading of: 1.49 mg (a) 1.35 mg (b).

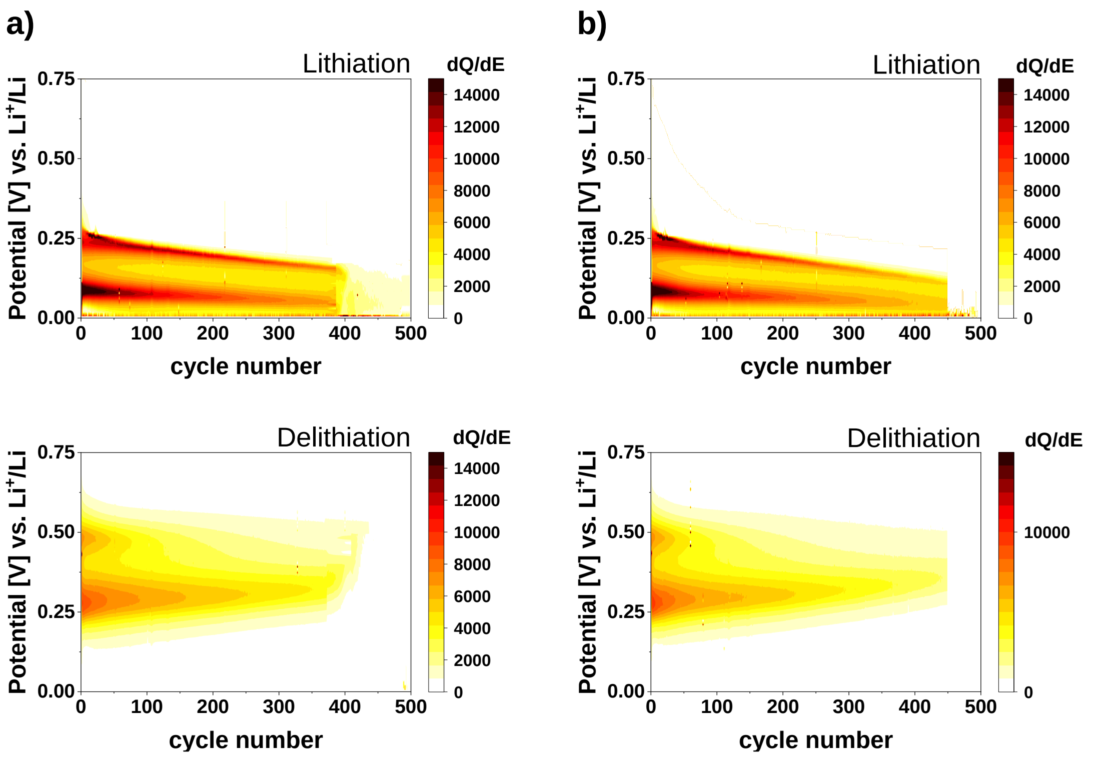

Figure S14. Heatmaps of dQ/dE vs. potential and cycle number for lithiation and delithiation of untreated Si electrodes with a loading of: 1.61 (a) 1.50 mg (b).

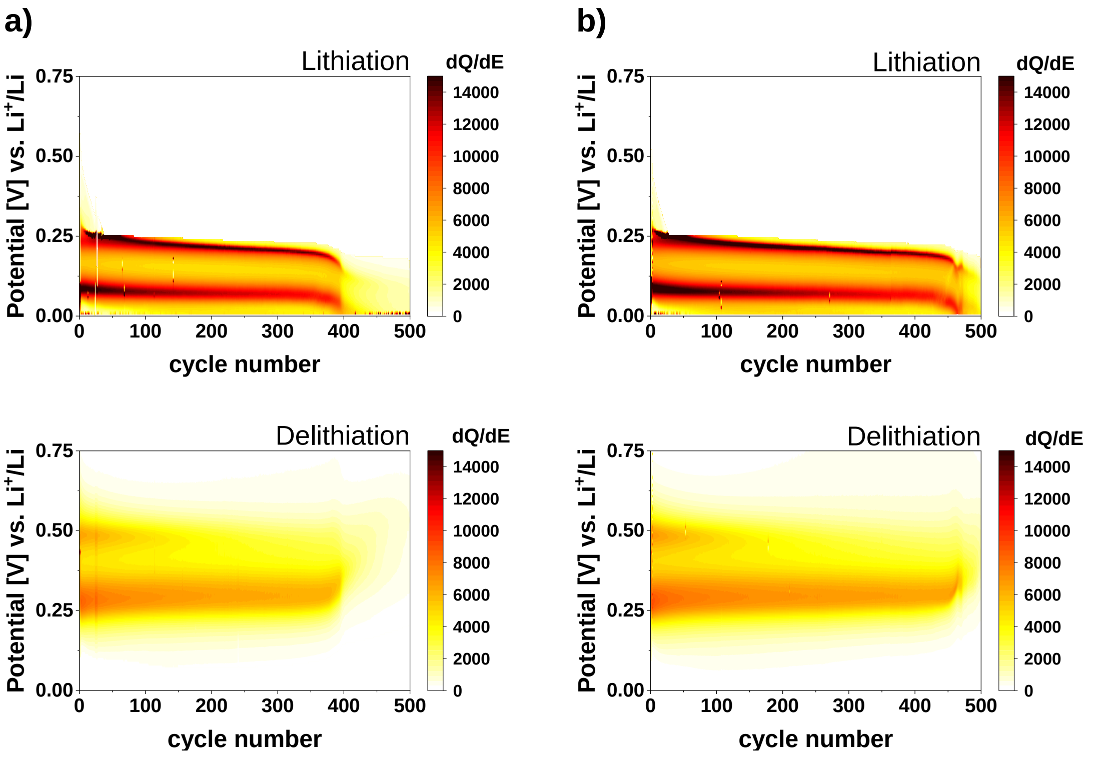

Figure S15. Heatmaps of dQ/dE vs. potential and cycle number for lithiation and delithiation of TMA/ethane-1,2-diol treated electrodes with a loading of: 1.47 mg (a) 1.36 mg (b).

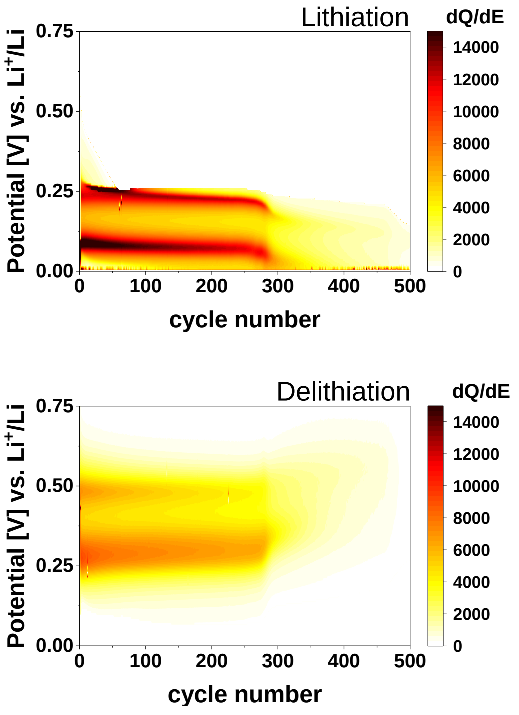

Figure S16. Heatmaps of dQ/dE vs. potential and cycle number for lithiation and delithiation of TMA/hexane-1,6-diol treated electrodes with a loading of: 1.62 mg.

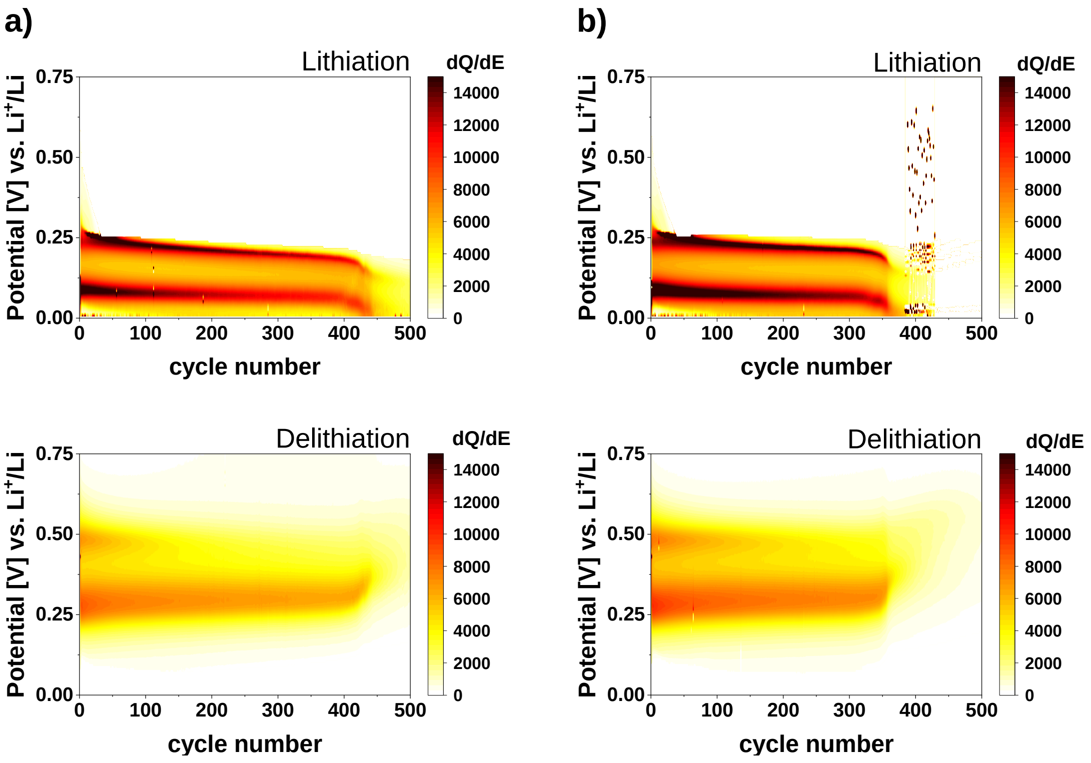

Figure S17. Heatmaps of dQ/dE vs. potential and cycle number for lithiation and delithiation of TMA/decane-1,10-diol treated electrodes with a loading of: 1.40 mg (a) 1.54 mg (b).

4. XPS supplementary data

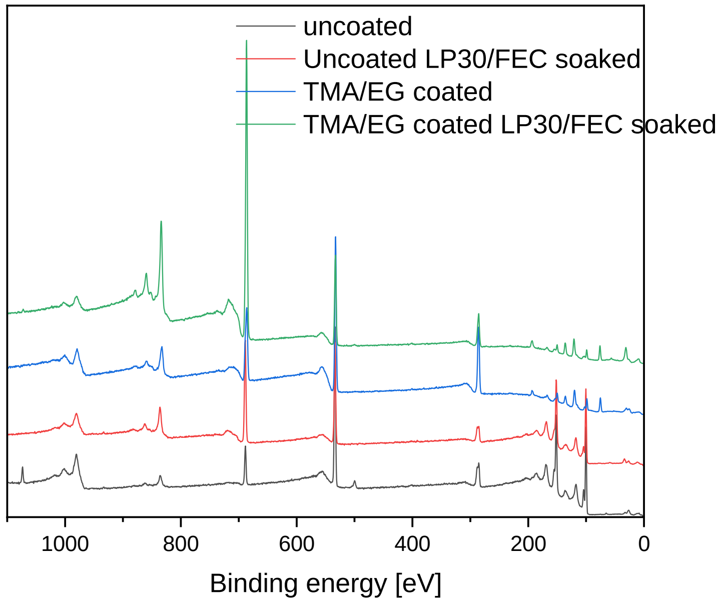

Figure S18. XPS surveys of 1st series of the analysed electrodes.

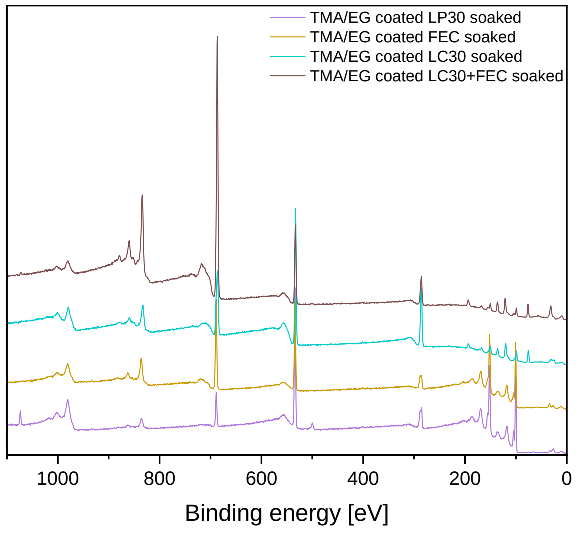

Figure S19. XPS surveys of 2nd series of the analysed electrodes.

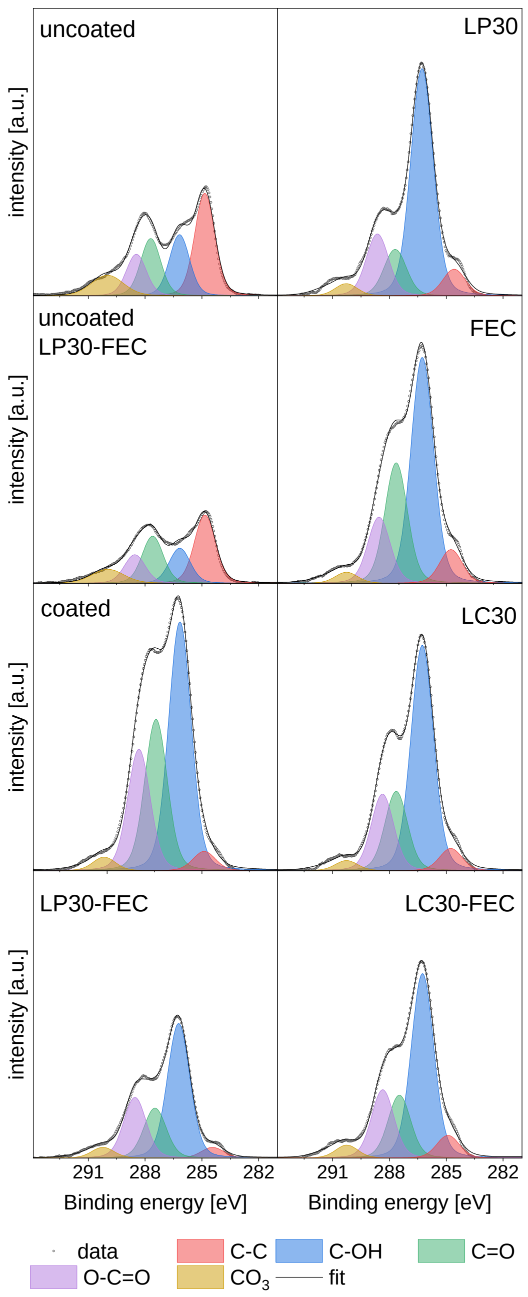

Figure S20. C 1s spectra of all the analysed samples.

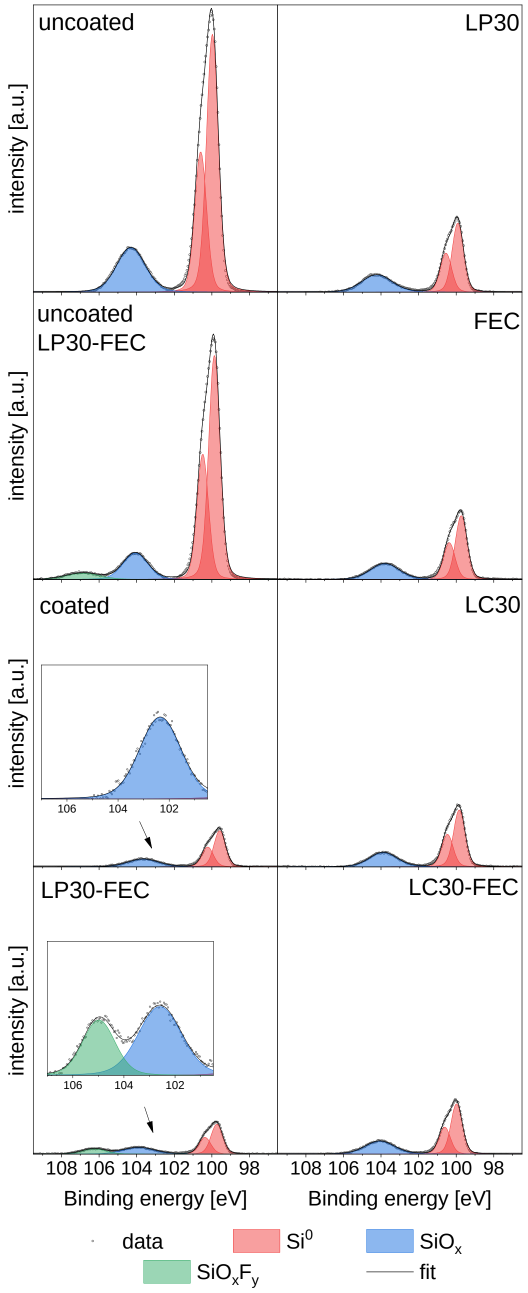

Figure S21. Si 2p spectra of all the analysed samples.

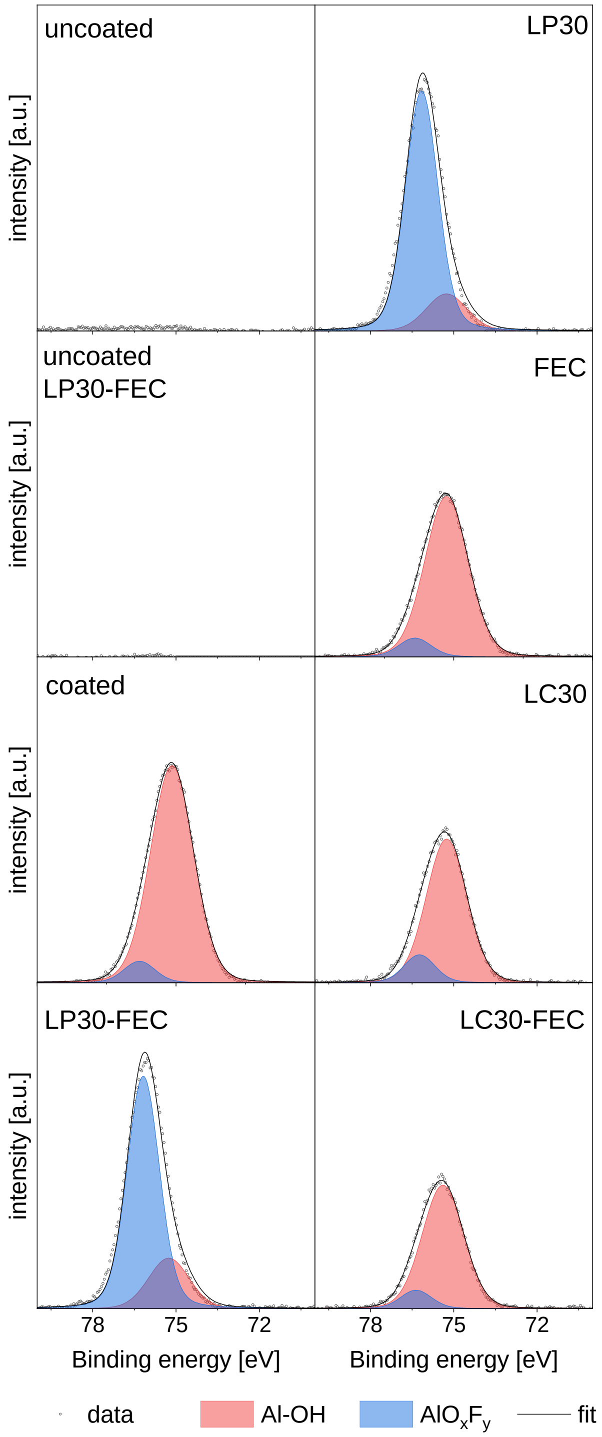

Figure S22. Al 2p spectra of all the analysed samples.

Table S2. Fit parameters for carbon.

	**Unocated**		**Uncoated + soaked**		**Coated**		**Coated + soaked**	
	BE [eV]	fwhm	BE [eV]	fwhm	BE [eV]	fwhm	BE [eV]	fwhm
**C-C**	284.85	1.3	284.84	1.3	284.83	1.3	284.85	1.4
**C-O**	286.18	1.3	286.17	1.3	286.13	1.3	286.30	1.2
**C=O**	287.7	1.3	287.6	1.3	287.30	1.3	287.16	1.3
**O-C=O**	288.46	1.3	288.54	1.3	288.27	1.3	288.59	1.4
**CO_3**	289.99	2.0	289.96	2.0	289.99	2.0	290.22	2.1

Table S3 Fit parameters for Al 2p.

	**Coated**			**Coated+ soaked**		
	BE /eV	fwhm	$\%_{at}$	BE /eV	fwhm	$\%_{at}$
**Al-(OH)**	75.2	1.8	93.1	75.3	1.7	20.9
**AlO_xF_y**	76.3	1.3	6.9	76.2	1.4	79.1

Table S4 Fit parameters for F.

	**Uncoated**			**Uncoated LP30/FEC soaked**			**TMA/EG coated**			**TMA/EG coated LP30/FEC soaked**		
	BE /eV	fwhm	$\%_{at}$	BE/eV	fwhm	$\%_{at}$	BE /eV	fwhm	$\%_{at}$	BE /eV	fwhm	$\%_{at}$
**$LiP_xO_yF_z$**	-	-	-	689.4	1.84	48.2	-	-	-	689.3	1.23	3.9
**SiO_xF_y**	-	-	-	688.4	1.84	51.8	-	-	-	688.6	1.18	4.9
**AlO_xF_y**	-	-	-	-	-	-	686.1	2.8	79.8	686.6	2.19	91.2

Table S5. Quantification of the elements detected on the surface of TMA/EG-treated electrodes immersed in different types of electrolytes.

	**Atomic %**			
**Element**	**LP30**	**FEC**	**LC30**	**LC30+FEC**
**Si 2p**	9.6	12.4	11.31	10.7
**C 1s**	22.8	36.2	35.3	32.6
**O 1s**	15.7	27.1	29.1	31.0
**F 1s**	33.8	14.0	12.5	12.5
**Na 1s**	0.2	0.1	0.2	0.2
**Li 1s**	9.5	-	1.6	2.8
**Al 2p**	8.5	9.3	8.8	8.5
**Cl 2p**	-	-	1.2	2.1
